# An error-tuned model for sensorimotor learning

**DOI:** 10.1371/journal.pcbi.1005883

**Published:** 2017-12-18

**Authors:** James N. Ingram, Mohsen Sadeghi, J. Randall Flanagan, Daniel M. Wolpert

**Affiliations:** 1 Department of Engineering, University of Cambridge, Trumpington Street, Cambridge, United Kingdom; 2 Department of Psychology and Centre for Neuroscience Studies, Queen’s University, Kingston, ON, Canada; Johns Hopkins University, UNITED STATES

## Abstract

Current models of sensorimotor control posit that motor commands are generated by combining multiple modules which may consist of internal models, motor primitives or motor synergies. The mechanisms which select modules based on task requirements and modify their output during learning are therefore critical to our understanding of sensorimotor control. Here we develop a novel modular architecture for multi-dimensional tasks in which a set of fixed primitives are each able to compensate for errors in a single direction in the task space. The contribution of the primitives to the motor output is determined by both top-down contextual information and bottom-up error information. We implement this model for a task in which subjects learn to manipulate a dynamic object whose orientation can vary. In the model, visual information regarding the context (the orientation of the object) allows the appropriate primitives to be engaged. This top-down module selection is implemented by a Gaussian function tuned for the visual orientation of the object. Second, each module's contribution adapts across trials in proportion to its ability to decrease the current kinematic error. Specifically, adaptation is implemented by cosine tuning of primitives to the current direction of the error, which we show to be theoretically optimal for reducing error. This error-tuned model makes two novel predictions. First, interference should occur between alternating dynamics only when the kinematic errors associated with each oppose one another. In contrast, dynamics which lead to orthogonal errors should not interfere. Second, kinematic errors alone should be sufficient to engage the appropriate modules, even in the absence of contextual information normally provided by vision. We confirm both these predictions experimentally and show that the model can also account for data from previous experiments. Our results suggest that two interacting processes account for module selection during sensorimotor control and learning.

## Author summary

Research in motor learning has focused on how we acquire new motor memories for novel situations. However, in many real world motor tasks, the challenge is to select appropriate memories for a given context. In such tasks, we are guided by two key types of information. First, contextual information from vision (for example) is available before we perform the task. Second, movement errors are available as we begin to perform the task. Here we present a model that provides a mechanism by which these two processes operate in parallel to enable us to tune and adapt our motor commands. We show that a model consisting of multiple simple modules, each of which can correct errors in a single direction only, can account for learning in multidimensional tasks. The model makes predictions about which tasks should interfere and how experience of errors alone without any contextual information can drive learning. We confirm these predictions in a series of experiments. The model provides a new framework for understanding the interaction between task context and error feedback during sensorimotor control and learning.

## Introduction

Current models of sensorimotor control posit that motor commands are generated by multiple modules which can be selectively engaged depending on the requirements of the task [1–6]. Familiar examples of modular architectures include multiple internal models [1,7–9], motor primitives [10–13] and motor synergies [14–19]. Within this framework, the mechanisms which select modules and modify their output are critical to our understanding of sensorimotor control. However, despite growing evidence for modularity from a variety of paradigms (for review see [20]), the details of module selection and learning remain poorly understood. Theoretically, two distinct but interacting processes for module selection have been proposed [1]. In the first process, information about the context of the task is used to engage the appropriate modules. For example, before an object is grasped, visual information about the object can be used to estimate the dynamics (for example, the centre of mass) and thereby apply appropriate control [e.g. 21, 22–24]. In the second process, errors occurring during a movement can be used to modify the contributions of the modules to the motor output for future movements. For example, once an object has been grasped and manipulated, movement errors can be used to update the module contribution so as to reduce errors when the object is manipulated again [e.g. 25].

A range of studies have focused on how bottom-up errors drive trial-by-trial adaptation and have used state-space models to capture motor learning in various tasks [10,26–28]. A number of studies have also incorporated the use of top-down contextual information [27,29]. However, in general this contextual information has consisted of differences in movement kinematics (for example, movements to different target locations). In contrast, contextual information during motor tasks can change the dynamics of the task without changing the movement kinematics (for example, differences in the size, shape and orientation of a hand-held object). Moreover, many current state-space models only deal with scalar error, whereas motor errors can be multi-dimensional. Here we examine the interaction of information derived from the visual context and kinematic error in a task in which both the context (the orientation of an object) and error direction (the displacement of the control-point on the object) can take on continuous values. We use a two-dimensional object manipulation task in which the desired movement kinematics are the same across differences in context and the error is a vector rather than a simple scalar value.

We develop a novel modular architecture in which each module is one-dimensional and therefore capable of generating force in only one direction (its preferred direction). Both the top-down context and bottom-up errors contribute to adaptation of modules across consecutive trials. Within a given trial, the motor output is determined by both the context and the current adaptive state of the modules. As well as reproducing results of experiments from previous studies, the model makes new predictions. Specifically, the model predicts that interference should occur between alternating dynamic contexts only when the kinematic errors associated with each context oppose one another. In contrast, dynamic contexts with orthogonal errors should not interfere. The model also predicts that the tuning of modules to particular errors is sufficient to engage the appropriate modules, even in the absence of contextual information normally provided by vision. Using our object manipulation paradigm we confirm both predictions.

## Results

**Fig 1:**
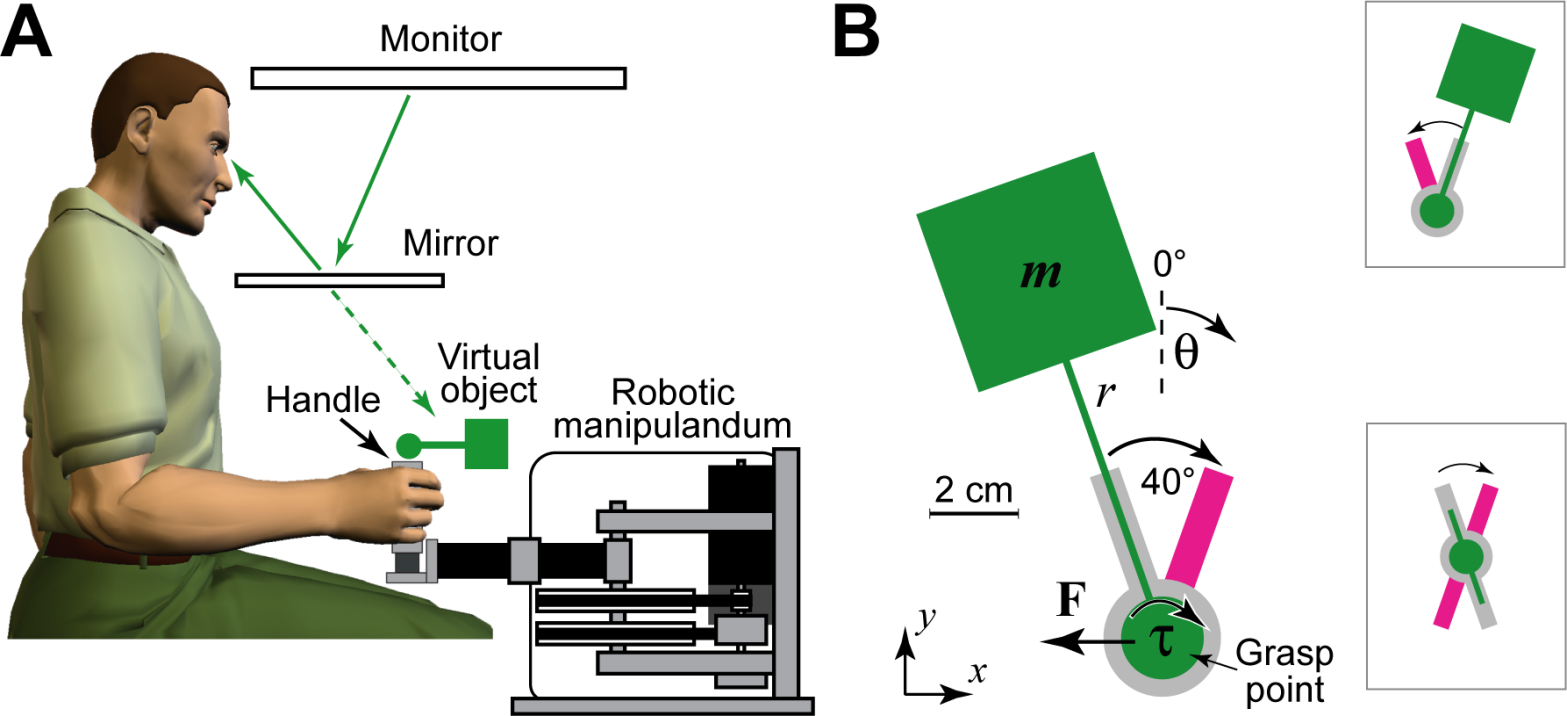
Object manipulation task. **A.** Subjects grasped the handle of a robotic manipulandum (the WristBOT) that could generate forces in the horizontal plane and torques around the vertical handle. A mirror-monitor system projected an image of the object and the task into the plane of the movement. **B.** Subjects rotated the object (green) clockwise and counter-clockwise (top inset) between visually presented targets (purple) and were required to keep the handle (grasp point) as still as possible within the central home region (grey). On exposure trials, the dynamics of the object (forces and torques) were consistent with rotating a mass (*m*) on the end of an 8 cm rod (*r*). Rotation of the object generates forces at the handle (**F**) that are approximately orthogonal to the orientation (θ) of the rod. In order to maintain the handle stationary while rotating the object, the subject must counteract these forces. The visual orientation of the object could be made ambiguous by presenting an ambiguous object (bottom inset). The rotation and translation of the visual object (normal or ambiguous) always tracked the rotation and translation of the WristBOT handle.

Subjects were required to rotate a hammer-like object clockwise (CW) and counter-clockwise (CCW) while maintaining the handle stationary (Fig 1A and 1B). They grasped the handle of a robotic manipulandum (the WristBOT [30]) which simulated the torques and forces associated with rotating the object. On each trial, both the visual orientation and dynamics of the object could be varied (Fig 1B). Specifically, across the trials in each experiment, the object could be presented at different orientations relative to the hand. In addition, the dynamics simulated by the WristBOT could vary according to three different trial types. The trial types consisted of exposure trials (torques and forces associated with the full object dynamics), zero-force trials (object torques only with the handle free to translate) and error-clamp trials (object torques with handle translation clamped by a simulated spring). Importantly, on exposure trials, to prevent the handle from displacing, subjects had to generate compensatory forces to oppose the forces associated with the circular motion of the mass. We focus on two measures in the task. On exposure and zero-force trials, we measure kinematic error as the peak displacement (PD) of the handle. On error-clamp trials, we measure the force adaptation as the ratio of the peak force generated by the subject to the peak force that the object would have generated on the equivalent exposure trial.

### The error-tuned model (ETM)

**Fig 2:**
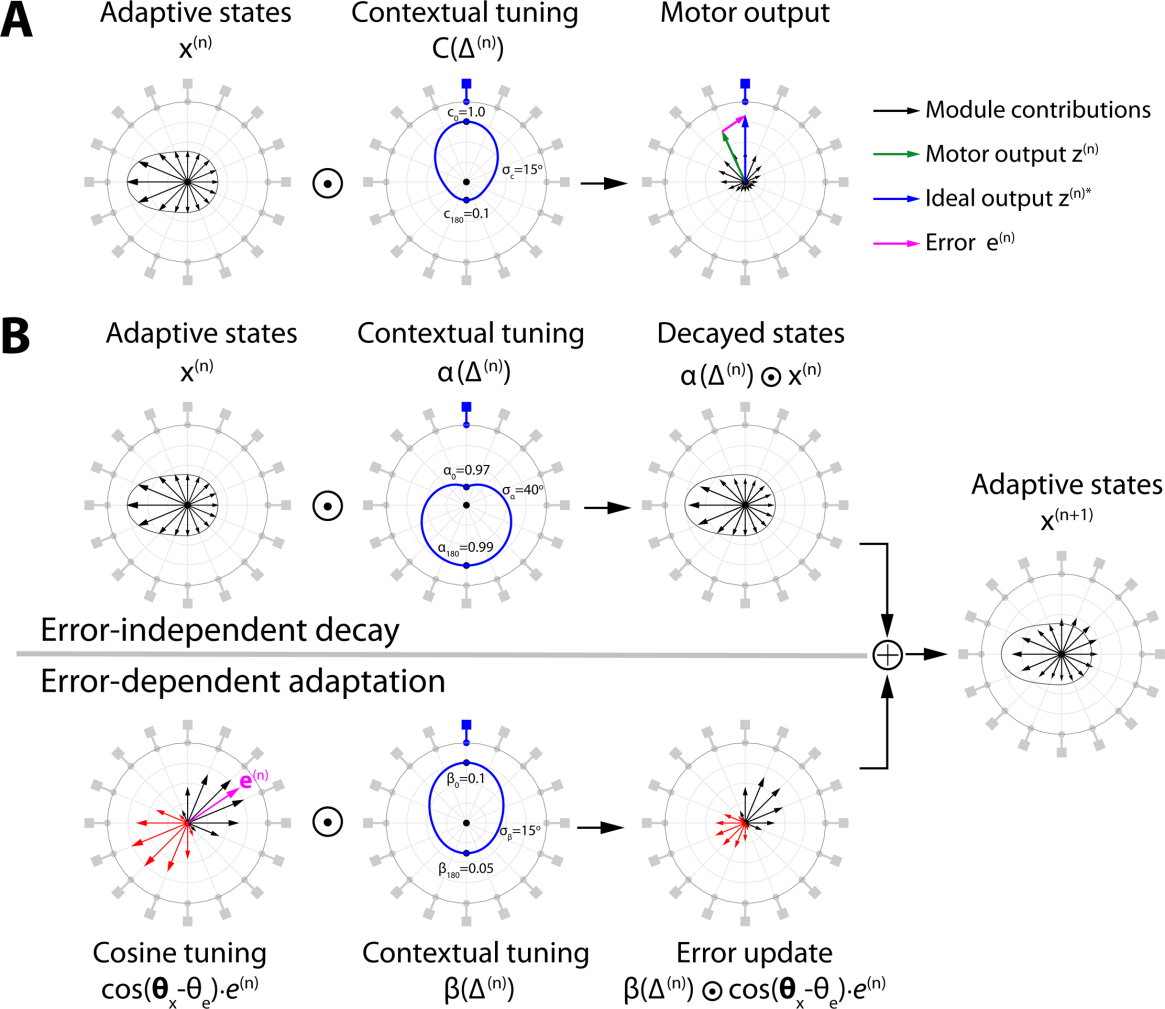
Schematic of the error tuned model (ETM). **A**. Motor output. The modules each have a preferred direction uniformly covering the possible object orientations (here, 16 modules are shown by the grey peripheral objects). On the n^th^ trial the modules each have an adaptive state indicated by the length of the vectors (left panel). In this example, the distribution of adapted states is consistent with recent experience of an object at 270°. On the current trial, the object is changed to an orientation of 0° (blue peripheral object). In this case, the visual contextual tuning gives the greatest weight to modules with preferred directions near 0° (middle panel). The motor contribution of each module (black vectors, right panel) is vector summed to produce the final motor output (green vector). The ideal motor output is shown by the blue vector, leading to an error (magenta vector). **B**. Motor adaptation is driven by two processes. The top row shows error-independent decay in which visual contextual tuning (middle panel) determines the decay of memory across modules. Here the memory decays most for the current context (0°) and less for more distant contexts. This leads to a set of reduced adaptive states (right panel; original states indicated by solid line). The bottom row shows error-dependent adaptation. The left panel shows the error (magenta) as well as its projection onto each module’s preferred direction (i.e. cosine tuning in which red vectors reflect negative magnitudes). This tuning reflects the extent that changing the adaptive state of a module will reduce the error. These projections are modulated by the visual contextual tuning (middle panel) which is greatest for the current context. This determines how each module updates its adaptive state in response to the error (right panel). The adaptive state on the next trial (n+1; far right panel) is the sum of the decayed states and the state updates, leading to a reduced error on the next trial for the same orientation of the object. Note that this schematic is not drawn to scale and exaggerates some of the changes so that they are visible. The ⊙ symbol represents element-wise multiplication across the modules.

We developed a novel error-tuned model (ETM; Fig 2) to examine the interaction of context (visual object orientation) and kinematic error (handle displacement) during adaptation in our task (for full details see Methods). The ETM is a state-space model which consists of *m* modules. Each module can generate a force in a single fixed preferred direction (*θ*_*i*_ for the *i*^*th*^ module). The state of each module ($x_{i}^{\left(n\right)}$ on the *n*^*th*^ trial) represents its level of adaptation (Fig 2A, left panel). The adaptive state can change across trials and determines the magnitude of the force produced by that module on a given trial. The preferred direction across modules uniformly covers all directions in the two-dimensional task space.

The motor output (Fig 2A, right panel) on a given trial is the weighted vector sum across the activity of all the modules. The weighted contribution of each module is determined by both the adaptive state of that module ($x_{i}^{\left(n\right)}$) and the difference between the visual context (orientation) of the object ($\theta_{v}^{\left(n\right)}$) and the preferred direction of the module (*θ*_*i*_):

$$\Delta_{i}^{\left(n\right)}=\theta_{i}-\theta_{v}^{\left(n\right)}$$ (1)

The motor output (***z***^(*n*)^) is given by:

$$z^{\left(n\right)}=\sum_{i=1}^{m}C(\Delta_{i}^{\left(n\right)})x_{i}^{\left(n\right)}\;\left[\begin{array}{c} \cos\theta_{i}\\ \sin\theta_{i} \end{array}\right]$$ (2)

where the context-dependent weighting $C(\Delta_{i}^{\left(n\right)})$ is implemented as a Gaussian tuning function (Fig 2A, middle panel). Modules with preferred directions closest to the current context thus receive the highest weighting.

Errors in the model (right panel of Fig 2A) result from the discrepancy between the motor output (green vector) and the forces associated with the dynamics of the object (blue vector). Because both the motor output and the object dynamics are represented in a two-dimensional task space, the error on a given trial is also a two-dimensional vector (magenta vector). The magnitude (*e*^(*n*)^) and direction ($\theta_{e}^{\left(n\right)}$) of the error vector both contribute to adaptation in the model.

The adaptive state for the *i*^*th*^ module changes based on two processes (Fig 2B):

$$x_{i}^{\left(n+1\right)}=\alpha (\Delta_{i}^{\left(n\right)})x_{i}^{\left(n\right)}+\beta (\Delta_{i}^{\left(n\right)})\cos(\theta_{i}-\theta_{e}^{\left(n\right)})e^{\left(n\right)}$$ (3)

First, an error-independent process (Fig 2B, top row) causes trial-by-trial decay in the adaptive state. This decay is context-dependent and is implemented by a Gaussian tuning function ($\alpha (\Delta_{i}^{\left(n\right)})$; Fig 2B, top row, middle column). This determines the extent to which the adaptive state of each module is retained on the next trial. Second, an error-dependent process (Fig 2B, bottom row) causes the adaptive state of each module to change so that errors are reduced across successive trials (for the same dynamics). This error-dependent process combines two factors. The first factor is a context-dependent learning rate, which is implemented by a Gaussian tuning function ($\beta (\Delta_{i}^{\left(n\right)})$; Fig 2B, bottom row, middle panel). This ensures the greatest weighting for errors applies to modules with a preferred direction closest to the current context. The second factor is the projection of the error onto the preferred direction of each module (cosine term multiplied by error magnitude; Fig 1B, bottom row, left panel). This ensures that each module is updated in proportion to the degree to which changes in its adapted state can reduce the error on the next trial. We show that such cosine tuning for errors is optimally efficient in that it reduces the error using the smallest change in the overall adaptive states of the modules (see Methods). The 7 parameters in the ETM determine the shape of the three contextual tuning functions (middle row in Fig 2).

### The Context-dependent Decay Model (CDM)

The ETM differs from our previous Context-dependent Decay Model [4] in several respects. The CDM learns only the scalar magnitude of force, with the assumption that the force is always generated in the ideal direction (which compensates for dynamics of the object). As such, the CDM update rule is only sensitive to the difference in magnitude between the scalar force output and the scalar force generated by the dynamics. In contrast, the ETM is a vector-based model which is sensitive to both the magnitude and direction of errors. As mentioned above, the output of the ETM is a force vector and a critical requirement of the model is that the vector error is appropriately assigned across modules during adaptation. This is implemented by cosine tuning for error, which is absent in the CDM and leads to qualitatively different predictions between the CDM and ETM.

### Experiments and model fits

We first fit both the CDM and ETM (which have 6 and 7 parameters respectively; see Methods for details) simultaneously to three of our previously published experiments [3,4] (experiments 1, 2 and 3 in the current study; n = 12 in all experiments and conditions). Based on these fits, we compare the models and generate novel predictions for the ETM based on the parameters obtained. We test the predictions in two new experiments (experiments 4 and 5).

**Fig 3:**
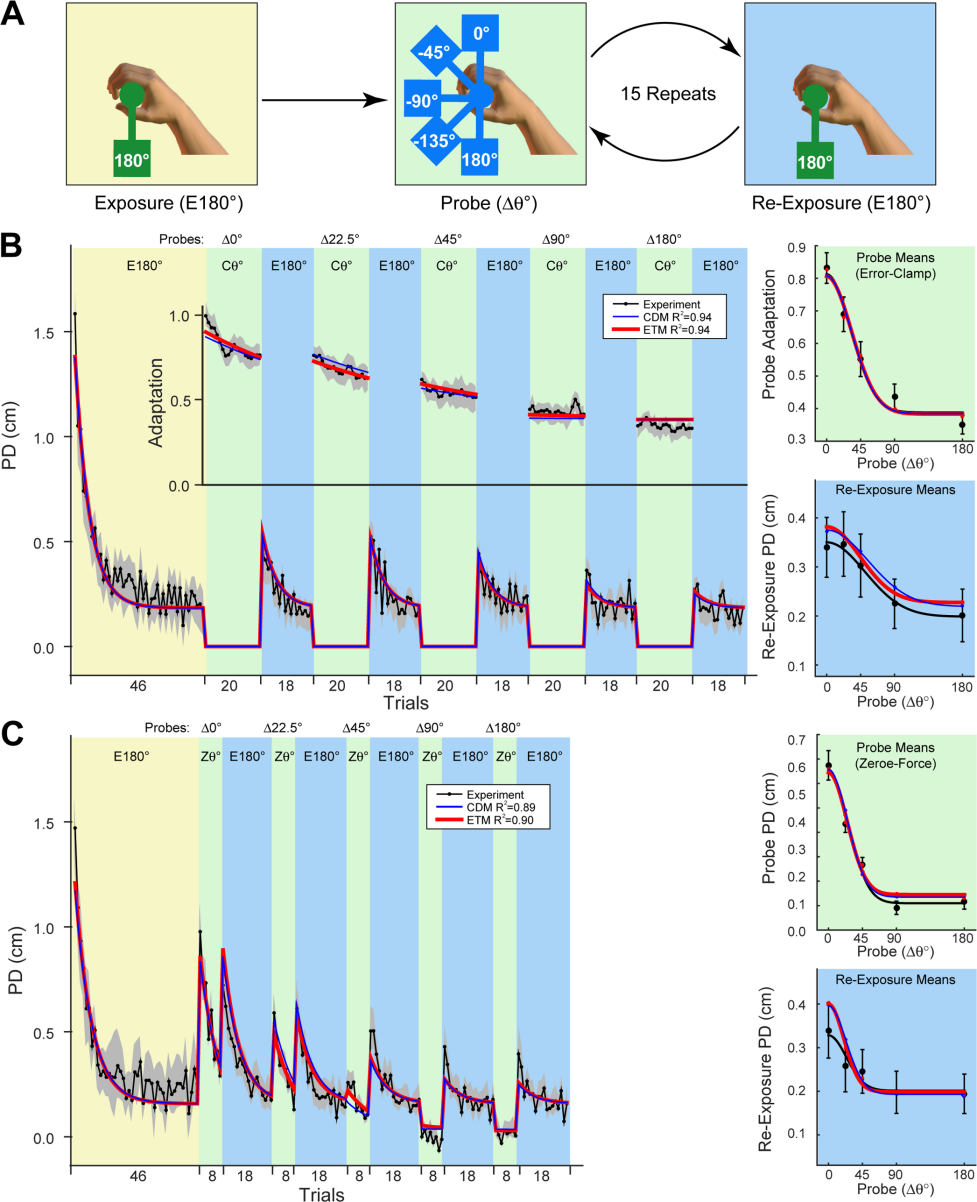
Experiments 1 and 2: Context-dependent adaptation and decay. **A**. The paradigm for experiments 1 and 2. After an initial exposure block at 180° (yellow background), subjects performed alternating probe blocks presented at one of five orientations between 0° and 180° (green background) followed by re-exposure blocks at 180° (blue background). **B**. Experiment 1 in which probe blocks consisted of 20 error-clamp trials. The left plot shows the composite trial-series for PD (all trials) and Adaptation (error-clamp probe blocks only). Grey shading shows ±SE across subjects. Each subject experienced the probe blocks in a pseudorandomized order so the trial-series has been rearranged in order of increasing probe orientation (∆0° to ∆180°). The right plots show the corresponding measures averaged over the different probe blocks and over subjects (error-bars show ±SE across subjects). Adaptation is measured from the probe blocks (right top, green background) and re-exposure PD is measured from the re-exposure blocks (right bottom, blue background). Model fits are shown in all panels for the CDM (blue) and ETM (red). Experimental data from [4]. **C**. Experiment 2, plotted as in panel B. In this case, probe blocks consisted of 8 zero-force trials. As in panel B, model fits are shown in all panels. Experimental data from [3].

The first two experiments (Fig 3) were designed to examine error-independent decay [4] and error-dependent adaptation [3]. In Experiment 1, subjects performed blocks of exposure at 180° which alternated with probe blocks consisting of 20 error-clamp trials presented across a range of orientations. The orientation of probe blocks was expressed relative to the exposure blocks (ranging from ∆0° to ∆180°; Fig 3A). For example, in the case of exposure at 180°, relative probe orientations of ∆0° and ∆180° represent error-clamp trials presented at 180° and 0°, respectively. Fig 3B shows the trial-series of peak displacement (PD) and adaptation (left) as well as the separate plots for the means across probe and re-exposure blocks, plotted for the different probe orientations (right). The mean adaptation measured during probe blocks is presented in the top-right plot (green background) and the mean re-exposure PD is presented in the bottom-right plot (blue background). Experiment 2 was similar except that probe blocks consisted of 8 zero-force trials. Fig 3C shows the results for Experiment 2 in the same format as Fig 3B.

These experiments provide evidence for both context-dependent adaptation and context-dependent decay. First, context-dependent adaptation was examined during probe blocks (green background in Fig 3) at a range of orientations relative to the exposure orientation. In both experiments, as the relative probe orientation increases (relative to the exposure orientation), the adaptation decreased progressively. Note that adaptation was measured by error-clamp trials in Experiment 1 (Probe Adaptation in Fig 3B) whereas PD was measured by zero-force trials in Experiment 2 (Probe PD in Fig 3C). This context-dependent pattern of adaptation is purely driven by the visual orientation of the object as the movement kinematics remain the same across all trials (see Methods). Second, context-dependent decay is evident in Experiment 1 during the performance on re-exposure blocks (blue background in Fig 3B). Re-exposure blocks occur immediately after each error-clamp probe block (re-exposure means in Fig 3B). During the error-clamp blocks, as errors are clamped to zero, the states of the modules undergo purely error-independent decay. During re-exposure, errors are greatest following error-clamp blocks presented near the original exposure orientation.

This pattern of context-dependent behaviour, whereby both learning and decay are greatest for the currently executed context, has been discussed in our previous paper [4]. Specifically, we suggest that the combination of decay and error-driven learning in the current movement context allows the motor system to constantly probe whether its force output is unnecessarily high while still maintaining low error. In contrast, the lower levels of learning and decay associated with more distant contexts allows these motor memories to be preserved.

Both the ETM and CDM (see Table 1 for model parameters and Supporting S1 Table for 95% confidence limits) can account for the context-dependent behaviours detailed above (see model fits in Fig 3; ETM red, and CDM blue). The same two experiments were also performed on separate groups of subjects who were exposed at 0° rather than 180° [3,4]. The models fits also included this data (see Supporting S1 Fig; R^2^ of CDM = 0.96 and ETM = 0.96 for exposure at 0° in Experiment 1; R^2^ of CDM = 0.82 and ETM = 0.85 for exposure at 0° in Experiment 2). See Supporting S2 Fig for 95% confidence limits on the ETM fits for both conditions (E180° and E0°) for Experiment 1 and Experiment 2.

**Table 1:** Model parameters. Parameters for the CDM and ETM when fit to datasets obtained from the different experiments. In the first dataset (top 2 rows for CDM and ETM), experiments 1, 2 and 3 were concurrently fit with all free model parameters. In the second dataset (bottom 2 rows for CDM and ETM), experiments 4 and 5 were concurrently fit with the Gaussian tuning function widths fixed to values obtained from fitting the first dataset (grey backgrounds indicate the fixed tuning-width values). BICs are relative to the best model within the fits for each dataset (the ETM in both cases). See Supporting S1 Table for 95% confidence limits.

Model	Exp	DoF	α_0_	α_180_	β_0_	β_180_	σ_α_	σ	C_180_	α_a_	β_a_	C_a_	R^2^	ΔBIC
CDM	1,2,3	6	0.9857	0.9981	0.1429	0.0001	50.8	31.1	-	-	-	-	0.87	122.0
ETM	1,2,3	7	0.9783	0.9960	0.1095	0.0225	38.9	14.5	0.0323	-	-	-	0.88	0.0
CDM	4,5	7	0.9731	0.9888	0.1826	0.0001	50.8	31.1	-	0.9895	0.0130	0.3769	0.88	287.3
ETM	4,5	8	0.9720	0.9957	0.1659	0.0222	38.9	14.5	0.1011	0.9895	0.0131	0.4285	0.90	0.0

**Fig 4:**
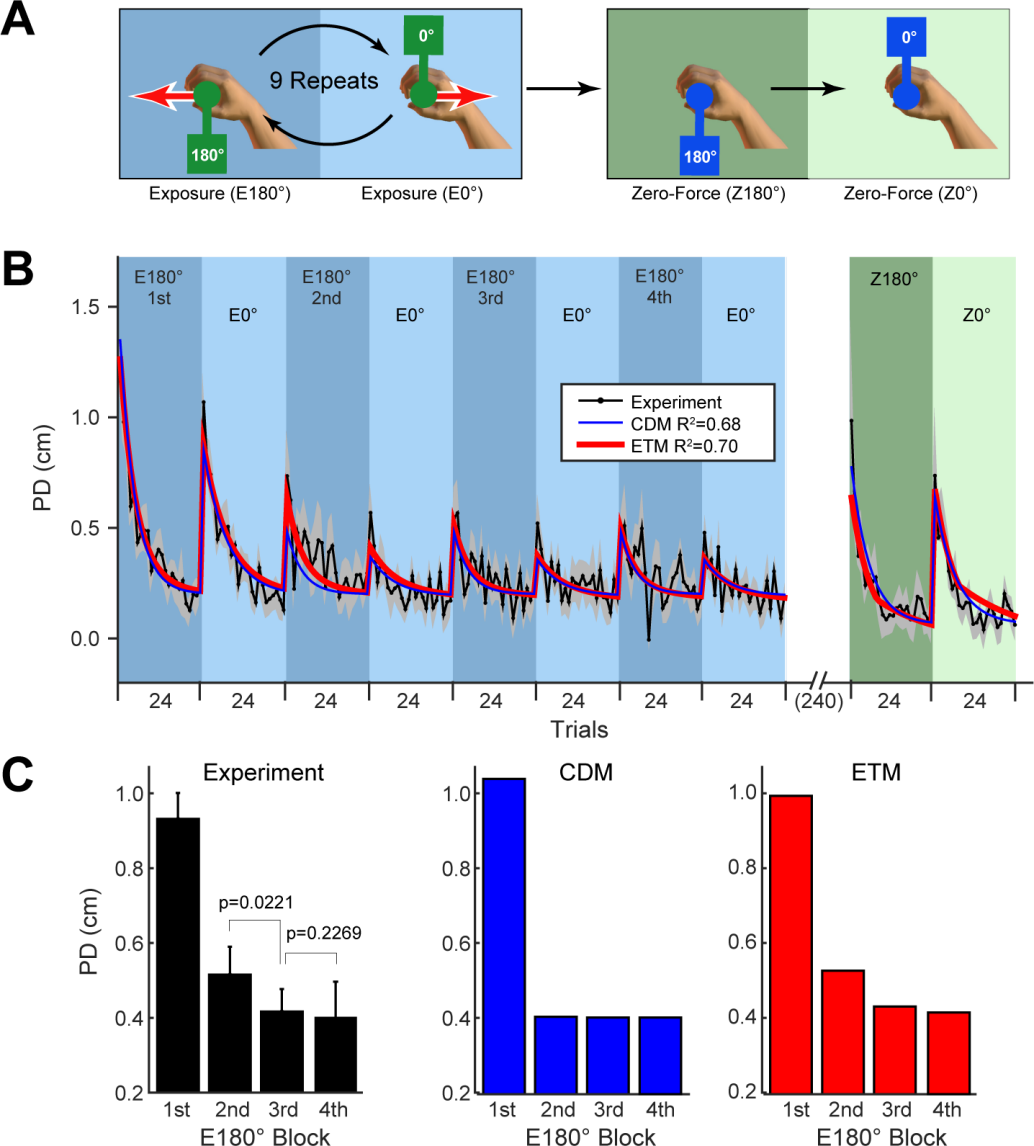
Experiment 3: Opposing dynamics. **A.** The paradigm consisted of alternating exposure blocks at 180° and 0° followed by two final blocks of zero-force trials (all blocks consist of 24 trials). **B.** Trial-series averaged across subjects (grey shading shows ±SE across subjects). Performance was stable from the 5th exposure cycle onwards so we omit exposure blocks after this for clarity. The fits of the models are shown in all panels for the CDM (blue) and ETM (red). **C.** The PD averaged over each of the first four 180° exposure blocks for the experimental data (error-bars are SE across subjects; p-values are for two-tailed paired t-tests as indicated) and CDM and ETM fits. Experimental data from [3].

Although both the CDM and ETM can reproduce the results of these first two experiments, they differ with regard to the third experiment. In Experiment 3 [3], subjects performed repeated alternating blocks of exposure trials at 180° and 0° followed by a final two blocks of zero-force de-adaptation trials (Fig 4A). Several important features are evident in the trial-series (Fig 4B including fits of CDM and ETM plotted as in Fig 3). During the first exposure blocks at each orientation, there are large errors which rapidly reduce. In addition, as the exposure blocks continue to alternate across the trials series, there is a small increase in error (and subsequent re-adaptation) at the start of each block. Finally, with regards to exposure blocks at 180° (the orientation experienced first), there is a progressive decrease in error across the first three blocks, with performance plateauing by block four (Fig 4C).

In the CDM, the increase in error between the end of one block at a particular orientation and re-exposure to that same orientation arises purely through error-independent decay. Importantly, however, as adaptation plateaus by the end of each block, any decay during the subsequent block will be the same across the experiment. Therefore, the fit of the CDM predicts that performance for the 180° blocks should plateau after the first exposure block (from the 2nd block; see Fig 4C blue). In contrast, in the ETM, the reduction in performance across consecutive blocks of the same orientation is not only driven by error-independent decay, but also by error-dependent interference between the opposing blocks. Because the force generated by the object for the two orientations (0° and 180°) are in opposite directions (see red vectors in Fig 4A), the kinematic errors will also tend to be in opposite directions. Therefore, adaptation during an exposure block at 0° will be partially driven by a reduction in the adaptive state of the module with a preferred direction of 180°. This reduction in the adaptive state of the 180° module arises because the error projected onto its preferred direction is negative (cosine tuning yields -1 in Eq 3). This reduces errors during the 0° block, but leads to increased error during the subsequent 180° block, as observed in the trial-series. Moreover, during the first two exposure blocks of the experiment, the adaptive state of modules corresponding to each orientation is zero. This leads to the large errors observed during these first two exposure blocks. Importantly, the large errors during the first 0° block causes substantial reduction in the adaptive state (interference) on the 2nd 180° block. Over consecutive blocks, this effect reduces (see experimental data plotted in Fig 4C). The ETM correctly predicts that the effects of interference should reduce across the first few 180° blocks. Therefore, unlike the CDM (blue plot in Fig 4C), the ETM is able to reproduce the progressive reduction of errors across 180° blocks observed in the data (red plot in Fig 4C).

In addition to these qualitative differences in the predictions of the CDM and ETM in Experiment 3, a BIC model comparison (see Methods) across all three experiments selects the ETM over the CDM (∆BIC = 122.0). See Supporting S2 Fig for 95% confidence limits on the ETM fits for Experiment 3.

### Interference and facilitation

**Fig 5:**
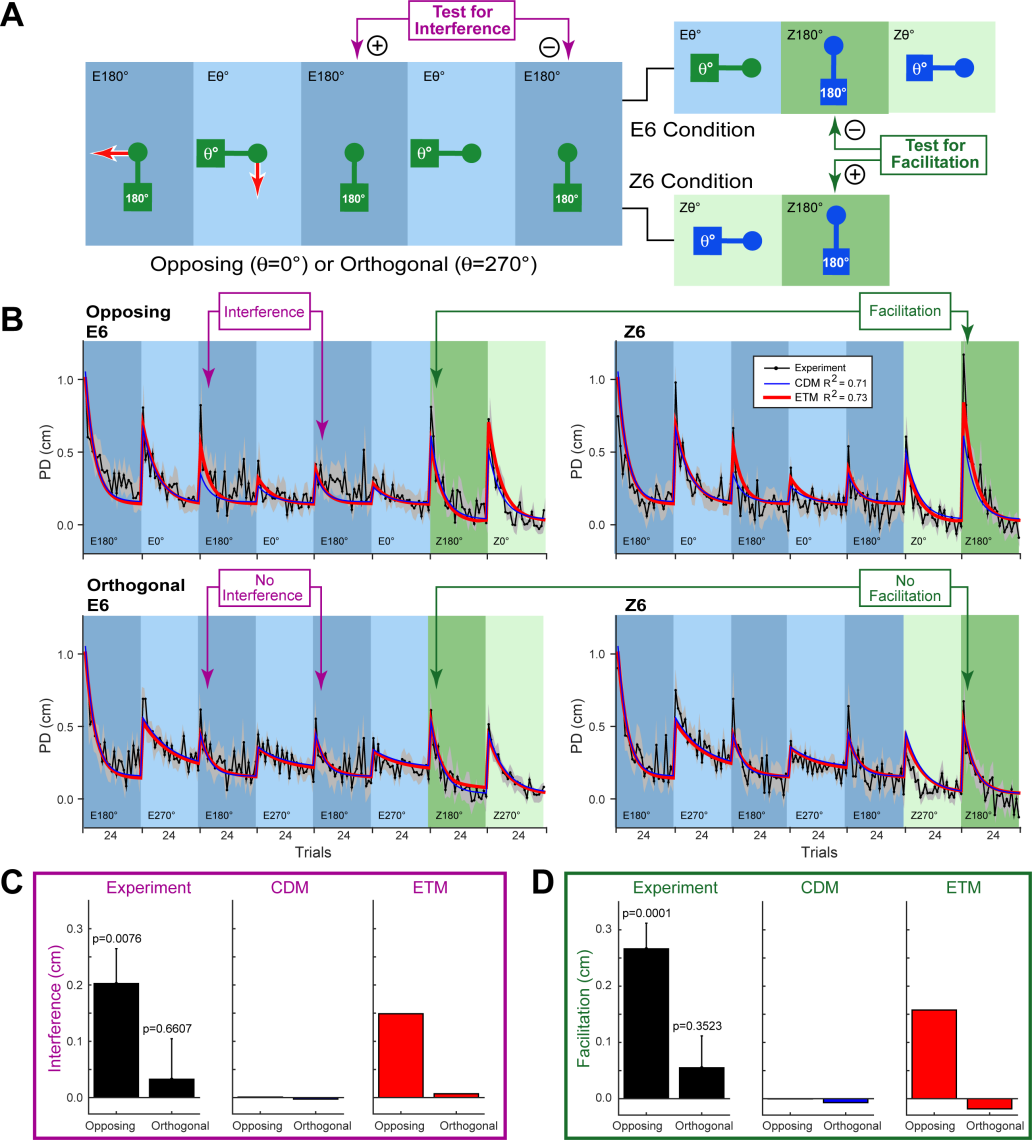
Experiment 4: Opposing versus orthogonal dynamics. **A.** The paradigm consisted of the first five blocks alternating between exposure at 180° and exposure at either 0° or 270° (opposing or orthogonal conditions; here shown as 270°). Interference was assessed by comparing PD on the 3rd and 5th block (purple comparison). The sixth block was either exposure (E6) or zero-force (Z6). Facilitation was assessed by comparing PD on the 7th block (Z180°) between E6 and Z6 conditions (dark green comparison). **B.** The trial-series for the four conditions (grey shading shows ±SE across subjects). Rows are opposing versus orthogonal dynamics and columns are E6 (exposure trials on 6th block) or Z6 (zero-force trials on 6th block). The model fits are shown in all panels for the CDM (blue) and ETM (red). Purple and dark green arrows test for interference and facilitation, respectively. **C.** Interference for the opposing and orthogonal conditions for the experimental data (black; error-bars show SE across subjects; p-values are for two-tailed paired t-tests) and the two models (CDM in blue; ETM in red). **D.** Facilitation, plotted as in panel C.

The interference in Experiment 3 arises because the errors experienced on consecutive blocks are in opposite directions. As a consequence, the modules selective for these errors (180° and 0°) can both contribute to adaptation on a given block (by either increasing or reducing their adaptive state). However, if the forces generated by the object (and the associated kinematic errors) on consecutive blocks are orthogonal (see red vectors in Fig 5A for object orientations at 180° and 270°), the modules selective for these errors (180° and 270°) cannot jointly contribute to adaptation. In the ETM, this arises through the cosine tuning for error. Specifically, there should be no change in the adaptive state of the module with a preferred direction orthogonal to the current error. This predicts that the interference patterns observed for opposing dynamics in Experiment 3 will not occur for orthogonal dynamics.

In addition, the ETM makes a prediction with regards to de-adaptation on zero-force blocks. On a zero-force block, the errors (after-effects) will be in the opposite direction to those on an exposure block. Therefore, modules with the preferred direction associated with the de-adaptation errors will reduce their adaptive state (that is, they should de-adapt). Critically, however, the ETM predicts that modules with the opposite preferred direction will increase their adaptive state (because this also contributes to decrease de-adaptation errors). Therefore, zero-force trials at 0° should lead to facilitation (an increase) of adaptation for the 180° module. In contrast, zero-force trials at 270° should not facilitate adaptation of the 180° module, as the errors in this case are orthogonal.

We test the predictions of interference and facilitation in Experiment 4, in which subjects (n = 12) performed four conditions in a randomized order (Fig 5A). In all conditions, the orientation of the object alternated across successive blocks (as in Experiment 3). All odd-numbered blocks were at 180° and all even-numbered blocks were either opposing (0°) or orthogonal (270° as shown in Fig 5A). To examine interference, the first five blocks were exposure blocks and we compared the change in performance between the 2nd and 3rd exposure blocks at 180° (Fig 5A; purple comparison). To examine facilitation, we compared performance on the final 180° zero-force block when it was preceded by either an exposure block or a zero-force block (Fig 5A; green comparison). Subjects thus performed four conditions with two factors: opposing versus orthogonal dynamics and exposure (E6) versus zero-force (Z6) trials on the 6th block (Fig 5A).

The full trial-series for the four conditions and fits for the two models are shown in Fig 5B (see Table 1 for model parameters and Supporting S1 Table for 95% confidence limits). The results show significant interference (2nd versus 3rd exposure at 180°) for the opposing but not orthogonal dynamics (see purple comparisons in Fig 5B and black bar plot in Fig 5C). The results also showed significant facilitation for the 180° zero-force block when this was preceded by a zero-force block at 0° (opposing) compared to an exposure block at 0° (see dark green comparisons in Fig 5B and black bar plot in Fig 5D). Note that because facilitation is measured on zero-force trials, it is also manifest as an increase in PD. As predicted by the ETM, no facilitation was seen for the orthogonal condition. Analysis of the same measures for the fits of the model shows that the CDM predicts neither interference nor facilitation (blue plots in Fig 5C and 5D). In contrast, the ETM predicts both these effects observed in the data (red plots in Fig 5C and 5D). A BIC model comparison across the four conditions in Experiment 4 also selects the ETM over the CDM (∆BIC = 115.2). See Supporting S3 Fig for 95% confidence limits on the ETM fits for Experiment 4.

### Visually ambiguous object

The two fundamental components of the ETM that affect adaptation are the contextual tuning functions (Fig 2A and 2B, blue curves, middle column) and the cosine tuning for kinematic error (Fig 2B bottom left). The contextual tuning functions determine how the visual orientation of an object influence both the motor output and the changes in the adaptive state (error-independent decay and error-dependent adaptation). However, the cosine tuning for error is independent of the visual orientation of the object. Therefore, the ETM predicts that when visual information about object orientation is absent or ambiguous, kinematic errors alone are sufficient to drive adaptation of modules with the appropriate preferred directions.

**Fig 6:**
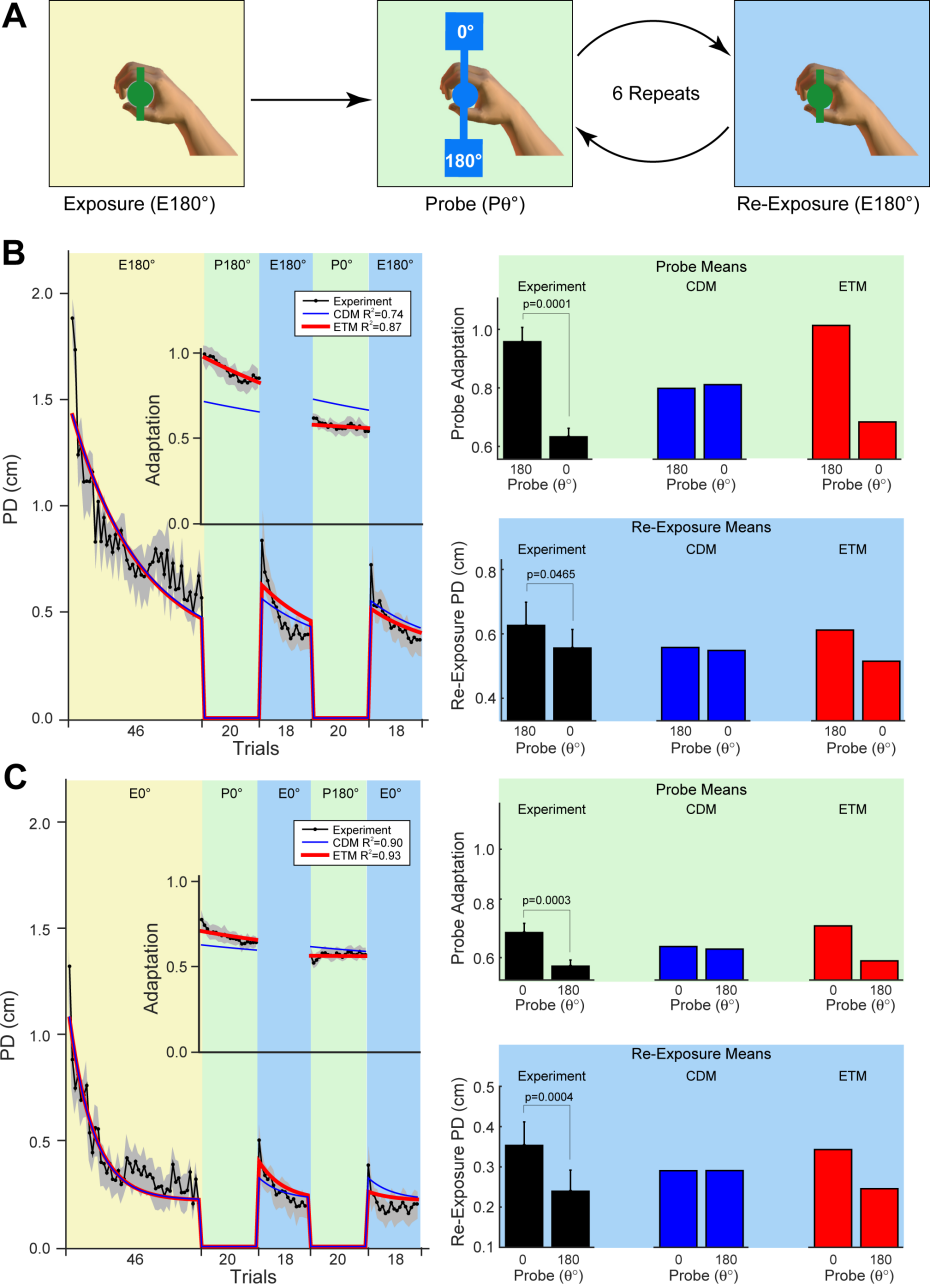
Experiment 5: Visually ambiguous object. **A.** The paradigm consisted of initial exposure to a visually ambiguous object with dynamics at 180° (yellow background), after which subjects perform alternating probe blocks (20 error-clamp trials) at one of two orientations (0° or 180°; green background) followed by re-exposure to the visually ambiguous object with dynamics at 180°. **B.** The right plot shows the composite trial-series for PD (all trials) and Adaptation (error-clamp probe blocks only). Grey shading shows ±SE across subjects. Each subject experienced the probe blocks in a pseudorandomized order. The trial-series has been rearranged in order of probe orientation (∆0° and ∆180°). The right plots show the corresponding measures averaged over the different probe blocks and over subjects (error-bars show SE across subjects; p-values are for two-tailed paired t-tests as indicated). Adaptation is taken from the probe blocks (right top, green background) and re-exposure PD is taken from the re-exposure blocks (right bottom, blue background). The model fits are shown in all panels for the CDM (blue) and ETM (red). **C.** A second group of subjects was exposed to the visually ambiguous object with dynamics at 0°. Results are plotted as in panel B.

In Experiment 5, we examine adaptation in the absence of contextual information by presenting subjects with a visually ambiguous object (Fig 1B bottom inset; Fig 6A). Subjects (n = 12) performed blocks of exposure trials at 180° which alternated with probe blocks of error-clamp trials with orientations of 0° and 180°. Critically, during the exposure blocks, the normal object dynamics were simulated (exposure at 180°) and the visual appearance of the object was ambiguous with regards to its visual orientation (Fig 1B bottom inset; Fig 6A). In this case, when the contextual information normally provided by vision is ambiguous, the context-dependent tuning functions take on constant values across orientations (see Methods). Cosine tuning in the ETM predicts that during exposure to a visually ambiguous object, the kinematic errors experienced by subjects should still selectively adapt modules with the preferred direction appropriate for the dynamics. To test this, the normal visual orientation of the object (and the associated contextual tuning of the output) is restored during probe blocks. The cosine tuning for errors in the ETM predicts that adaptation measured during these probe blocks should be largest for 180° compared to 0°. Importantly, because the visual probe blocks consist of error-clamp trials, subjects do not experience the dynamics. Rather, the probe blocks allow us to probe what modules have adapted to the visually ambiguous dynamics.

The experimental trial-series and model fits (see Table 1 for model parameters and Supporting S1 Table for 95% confidence limits) are shown in Fig 6B (left plot). The results show that adaption is greatest for the probes at 180° compared to 0° (black bars for probe adaptation in Fig 6B, right top plot, green background), in agreement with predictions of the ETM (red bars in the same plot). In contrast, the CDM predicts that exposure to visually ambiguous dynamics should result in the same adaptation across all modules (blue bars in the same plot).

On re-exposure to the ambiguous object at 180°, PD was greatest after probe blocks at 180° compared to 0° (black bars for re-exposure PD in Fig 6B, right bottom, blue background). This arises through context-dependent decay and is also predicted by the ETM (red bars in the same plot). Specifically, during error-clamp probe blocks, the normal visual appearance of the object was restored. The model predicts that during such error-clamp trials, context-dependent decay should occur. Thus, if the appropriate modules have adapted to the visually ambiguous dynamics, context-dependent decay during probe trials should also be greatest when the visual context matches those dynamics. This leads to the difference in PD values between the 2 probe contexts in the subsequent re-exposure (re-exposure plots in Fig 6B).

A separate group of subjects (n = 12) were also exposed to the ambiguous object at 0° (Fig 6C; Results plotted as for 180° exposure in Fig 6B). In this case, adaptation and re-exposure errors were greatest for the 0° probes compared to 180°, as predicted by the ETM (see bar plots on the right of Fig 6C). In addition, a BIC model comparison across both the 180° and 0° groups selected the ETM over the CDM (∆BIC = 295.3). See Supporting S3 Fig for 95% confidence limits on the ETM fits for Experiment 5.

### Object lifting study

**Fig 7:**
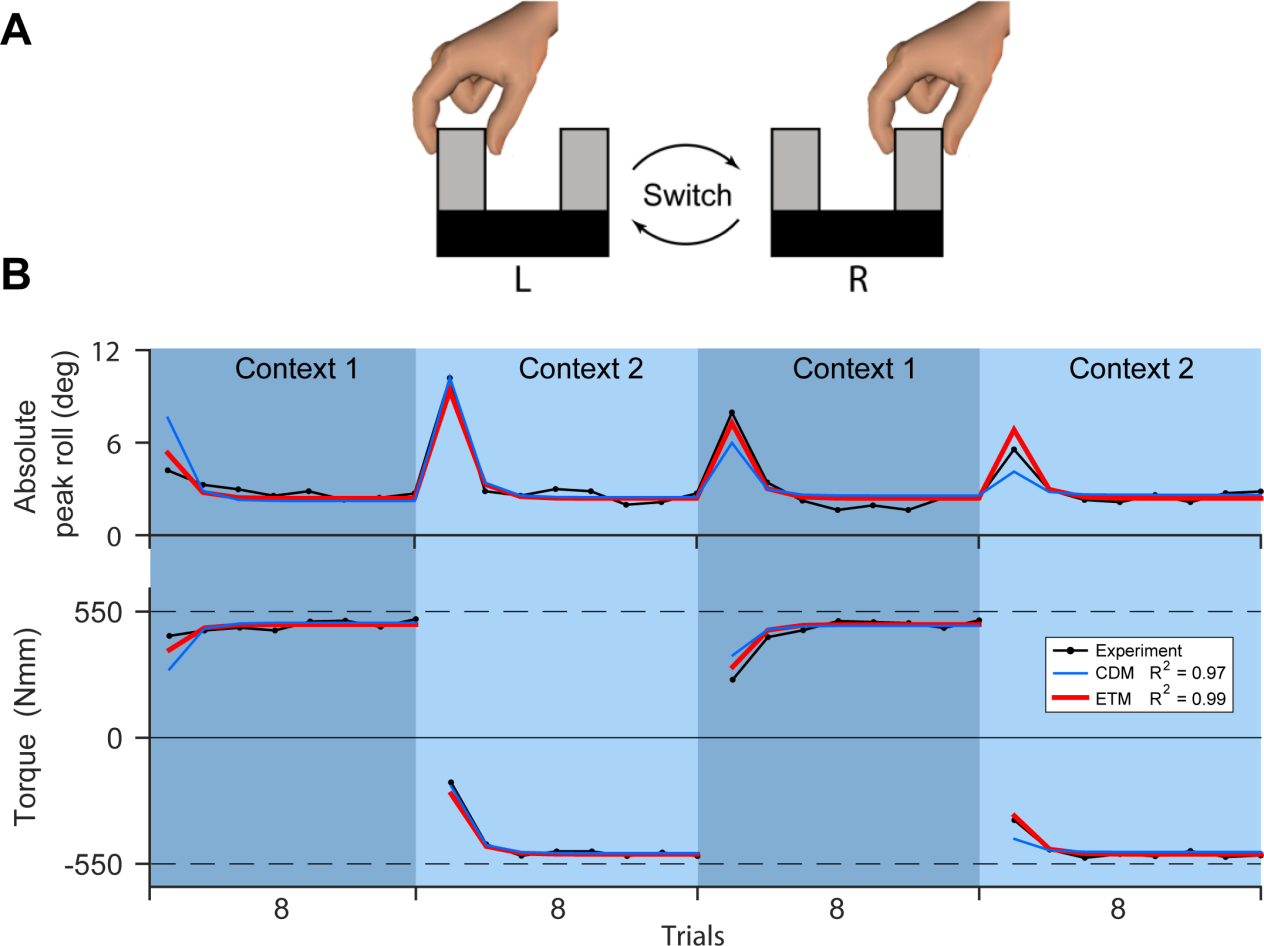
Object lifting experiment. **A**. The lifting paradigm used in [23]. Participants lifted a U-shaped object by alternating between the right-hand and left-hand grasp points in four blocks. **B**. The peak roll angle (tilt) of the object (top panel), as well as the compensation torque (bottom panel). Perfect compensation required ±550 N.mm depending on the context. Data is plotted in black and the fits for the ETM and CDM are plotted in red and blue, respectively. The best-fit parameters for the models (see Methods for details) are: *α*_0_ = 0.87, *β*_0_ = 0.74, *β*_180_ = 0.17, *c*_180_ = 0.0, *x*° = 0.66, and *k* = 15.68 for the ETM, and *α*_0_ = 0.92, *β*_0_ = 0.79, *β*_180_ = 0.00, *c*_180_ = 0.49, *x*° = 1.00 and *k* = 15.68 for the CDM.

A recent study of object lifting is particularly relevant for the ETM model [23]. In this study, subjects were presented with a U-shaped object that could be lifted by a handle on either the left (L) or right (R) side (Fig 7A). The task required subjects to lift the object from a table while minimising the tilt of the object. To do this, subjects had to apply compensatory torques on the handle as they lifted the object (a CCW torque for the left handle and a CW torque for the right handle). The visual location of the centre of mass relative to the grasp points is similar to the visual orientation of the object in our task. In the lifting study, subjects performed four consecutive blocks (8 trials each) with the context (lifting left or lifting right) alternating across blocks (Context 1 and Context 2). Task performance was measured as the peak tilt angle of the object during lifting and also the peak compensatory torque prior to object lift off [23].

When lifting the object for the first time, subjects used visual geometric cues about the object (the mass, centre of mass and location of the grasp point). This allowed them to lift the object with a small tilt angle and a torque close to that required for full compensation (first trial, Fig 7B). However, despite almost perfect performance by the end of the first block (Context 1), subjects showed significant errors when switching to the opposite handle (Context 2). These larger errors during the second block (for Context 2) suggest that interference is occurring (as observed in our experiments 3 and 4). Here, we show that the ETM can also account for this interference observed when lifting real-world objects.

We can consider the object lifting experiment within the same framework as the ETM model (see Methods). In this case, each module can generate a torque around a single fixed preferred oriented axis. The preferred torque axes across modules uniformly covers all orientations in the horizontal plane (corresponding to *θ*_*i*_ in the ETM). The context is the orientation of the centre of mass relative to the handle. As the original study only used two orientations (two grasp handles), we fit a simplified version of the ETM with only two modules (*θ*_*i*_ ∈ {0°,180°}) which correspond to producing CCW and CW torques for the left and right handles. In addition, we assumed that the initial adaptive state of both modules (*x*°) was non-zero and we include this as a fit parameter. This initial state represents existing knowledge of the dynamics of lifting objects, which may be based on visual cues.

The ETM successfully reproduces the experimental data for both absolute peak tilt angle and the compensating torque (Fig 7B, *R*^2^ = 0.99 across both measures). In particular, it reproduces the interference observed when switching from the first block (Context 1) to the second block (Context 2). It also reproduces the interference observed during re-exposure in the third and fourth blocks. In contrast, because the CDM does not model interference, it underestimates error during the third and fourth blocks (Fig 7B, *R*^2^ = 0.97).

## Discussion

We have developed a novel state-space model, the error-tuned model (ETM), which is based on a modular architecture. Each module produces force in a particular direction (its preferred direction) and can contribute to the net motor output in the two-dimensional task space. Modules do not change their preferred direction, but rather change their adaptive state, which determines their contribution to the net motor output. Critically, adaptation in the model (changes in the adaptive state across modules) is influenced by two processes. First, top-down contextual information (the visual orientation of the object) selectively weights the contribution of modules to the motor output. Contextual information also determines the degree to which errors update the adaptive states of each module. Second, bottom-up information provided by the magnitude and direction of kinematic errors also influence the adaptive states across modules. Specifically, each module is tuned to a particular error (determined by its preferred direction), and can change its adaptive state in proportion to its ability to correct the error. This selectivity for errors is implemented by cosine tuning for error direction.

The presence of error-tuned modules in the ETM makes two predictions which we confirm experimentally. First, cosine tuning predicts that dynamics which generate opposite kinematic errors will interfere. This arises because adapting to dynamics which cause kinematic errors in one direction will be partially achieved by reducing the activity (cosine tuning of -1) of modules appropriate for the opposite direction. In contrast, dynamics that generate orthogonal errors should show little interference. This arises because the cosine tuning for orthogonal errors (cosine tuning of 0) does not change the activity of the associated modules. We confirm these predictions, showing that interference is eliminated for orthogonal dynamics (Fig 5). Second, because errors can select the appropriate modules for update, the model predicts the selective adaptation of appropriate modules even in the absence of visual context. Using a visually ambiguous object, we show that the appropriate modules can adapt based on kinematic error in the absence of contextual information (see Fig 6). Together with the ability to account for previous data, these new results support a modular error-tuned model of motor learning.

Evidence for modularity in sensorimotor control has come from a variety of studies [1–6]. However, the details of module selection remain poorly understood. For example, studies of motor synergies have largely focused on extracting the patterns of muscle activity which underlie each synergy whereas the mechanisms by which different synergies are selected for a particular movement have received less attention [14–19]. Similarly, studies of motor learning have commonly focused on the acquisition of new internal models whereas less is known about the mechanisms by which these newly acquired models are selected from existing models [1,7–9]. As a result, the majority of computational models which include module selection rely on a single simplified mechanism [10,29]. In most cases, these mechanisms do not distinguish between kinematic factors (such as different movement directions) and contextual factors (such as the different states of a manipulated object). Moreover, the role of movement error in module selection, although suggested by theoretical studies [8], has received little experimental attention. In contrast, the ETM presented in the current study combines two mechanisms for module selection. As well as contextual information, which is the only mechanism considered by most current models, the ETM includes an error-based selection mechanism (cosine tuning for error).

The concept of motor primitives has been previously used in sensorimotor learning models mainly to study adaptation of reaching movements under state-dependent force-fields [12]. In these studies, primitives were defined in position and velocity space and were fit to experimental data to study biases seen when learning different types of force fields. A recent study [5] proposed a model based on a different form of motor primitives to explain how prospective (i.e. predicted) errors drive sensorimotor learning. In this model, primitives are defined in error space and are activated based on the size and sign of the prospective errors through a Gaussian function. The proposed model could explain several features of motor learning. However, the domain of predictions was limited to tasks with a one-dimensional error space and under a single context (for example, reaching in a single direction). In contrast, the primitives in the ETM represent force production in a two-dimensional space and they directly contribute to the motor output. In addition, the ETM primitives are activated jointly by two separate mechanisms; one based on the visual context of the environment, and the other based on the two-dimensional kinematic error.

The ETM and the previous models [3,4] that we have fit to our object manipulation task are all single-rate state-space models. These models have a single fast rate of adaptation which we assume is associated with a single adaptation process. Previously, we have speculated [3,4] that this fast single-rate adaptation process may be engaged when familiar dynamics are encountered (for example, the dynamics of everyday objects). In contrast, dual-rate state-space models have been applied to tasks in which the dynamics are novel and unfamiliar [26]. Dual-rate models assume that adaptation is mediated by distinct processes with different rates of adaptation. For example, these processes appear to map to different (explicit versus implicit) learning strategies [31] and appear to be independently affected by aging [32]. The relationship between the dual-rate adaptation processes examined in these previous studies and the single-rate process which mediates adaptation in the ETM are currently unknown. Further theoretical and experimental work would be required to elucidate this matter.

Interference has been observed across a range of motor tasks [33–37]. The demonstration of interference during object lifting is of particular relevance to the current study [23]. In our current study, the task required subjects to rotate an object while preventing it from translating, whereas in the object lifting task, subjects are required to translate (lift) an object while preventing it from rotating (tilting) by applying an appropriate torque [23]. In both cases, visual information about the context of the object as well as the direction and magnitude of kinematic errors can potentially contribute to adaptation across successive trials. In the ETM, these sources of information are combined in order to update the adaptive states of an array of modules which have different preferred directions of force output. Interference arises as a natural consequence of this modular architecture. Specifically, interference arises due to the interaction between errors and module output, but only when these are in opposite directions. Therefore, the ETM can model interference in the object rotation task and in the previous object lifting task (Fig 7). Importantly, in the current study, the ETM also correctly predicted that the interference observed for opposing dynamics should be reduced for orthogonal dynamics (Fig 5).

As discussed above, in the ETM, interference is a natural consequence of the interaction between modules with opposite preferred directions. An alternative model has previously been proposed to explain interference during object lifting [38]. In this model, interference between the modules producing CW and CCW torques does not arise due to the interaction between modules with opposite preferred directions (as it does in the ETM). This is because the terms which mediate this interaction have been specifically set to zero in the model. Rather, interference in this previous model is captured by a bias term which is added to the motor output. Our simulations show that if modules with opposite preferred directions are allowed to interact (with non-zero interaction terms), this bias term becomes unnecessary to reproduce interference. Moreover, it is unclear whether the bias term could generalize to account for our results in two dimensions.

A number of studies have examined the role of contextual information in engaging sensorimotor modules prior to movement [21,39]. For example, studies have shown that contextual cues can reduce the interference that normally occurs between two opposing velocity-dependent force-fields [39–43]. These studies show that when contextual information is provided which distinguishes between the two fields, subjects can adapt their feed-forward motor commands to the opposing dynamics. These results provide evidence that contextual information is used to select between separate internal models of task dynamics prior to a movement. In contrast, the role of movement errors in engaging and updating sensorimotor modules is less well understood. Importantly for the modular framework of sensorimotor control, evidence that movement errors can selectively engage and modify the output of specific sensorimotor modules is also lacking. In the current study, we provide evidence that modules are indeed tuned to movement errors so as to respond appropriately depending on their ability to correct an error. In neurophysiological studies, similar tuning for the direction of movement errors has also been found at the level of single neurons [e.g. 44, 45].

In the ETM, the error-based selection of modules is mediated by a cosine tuning function. For a particular error, cosine tuning determines the extent to which each module should change its activity so as to reduce the error progressively in the following trials. In previous studies, cosine tuning functions have been used effectively in the analysis of various features of the motor system, from tuning curve characteristics of motor cortical cells [46] to determining the contribution of muscle activity in force generation [47]. It has also been shown that cosine tuning can be an optimal solution in some cases, such as minimizing the net effect of neuromuscular noise in force production [48]. Here, we show mathematically that cosine tuning of modules to the direction of error in the ETM leads to an optimal selection process akin to a minimum intervention principle (see Methods). We demonstrate that for a particular error, cosine tuning will reduce the error with the minimum possible change in the activity across the modules.

Although the ETM is a high-level computational model, it is possible to speculate about its neural implementation in the brain. For example, there are three critical components of the ETM and these may be associated with known neural mechanisms. First, modules in the ETM have a preferred direction of force output and cells in primary motor cortex have also been found to have a preferred direction of action [46]. Second, contextual information about the visual orientation of the object mediates the top-down selection of modules in the ETM and the parietal cortex is thought to specifically encode object orientation when such information is required by the motor system [49]. Thirdly, modules in the ETM are tuned for the direction of kinematic errors and cells selective to error direction have been found in the cerebellum [50]. How these and other neural mechanisms are combined for the control of movement is an important topic for research.

The results of the current study suggest that sensorimotor modules are tuned to detect specific errors, changing their contribution to the motor output only if such a change is appropriate for reducing that particular error. We have demonstrated that such a process can account for adaptation seen when manipulating objects and also in a previous study of lifting objects. These were specific tasks which operated in a two-dimensional space. However, modular architectures have been implicated in a variety of different forms and across a variety of different tasks. The selective tuning of modules to particular errors may thus be a general feature of motor learning. In this view, across the array of possible modules which may be involved in a particular task, a central mechanism for motor learning is the ability of each module to detect those errors which are relevant to its output. Such error-tuning may be a general principle of sensorimotor control.

## Materials and methods

### Ethics statement

The study was approved by the Cambridge Psychology Research Ethics Committee. The subjects (a total of 96 university students) gave written informed consent before participating and were naïve to the purpose of the study.

### The object manipulation task

The object manipulation task has been previously described [3]. Briefly, subjects were seated at a virtual reality system and grasped the handle of a planar robotic manipulandum (the WristBOT) with their right hand (Fig 1A). The WristBOT [30] simulated the dynamics (forces and torques) of a hammer-like object which consisted of a mass on the end of a rigid rod (Fig 1B). Subjects grasped the object by a handle at the base of the rod (the grasp point in Fig 1B).

The virtual reality display system provided visual feedback associated with the object and the task (Fig 1B). The visual object consisted of a circular handle (radius 0.5 cm) attached by an 8 cm rod (width 0.2 cm) to a 4 cm square mass. The task involved rotating the object 40° between two visually presented targets (0.6 x 2.5 cm oriented rectangles). Subjects made alternating clockwise (CW) and counter-clockwise (CCW) rotations across trials (Fig 1B) and were asked to maintain the handle as still as possible within a central home region (1 cm radius). The position and orientation of the object tracked the position and orientation of the WristBOT handle. The angular midpoint between the two targets defined the orientation of the object and this could vary across trials in the experiments (the targets were thus ±20° relative to the presentation orientation).

### The object dynamics

The dynamics of the object were simulated as a point mass (1% of the subject’s body mass) at the end of the rigid rod (Fig 1B). Rotating the object generated forces and torques at the handle which were simulated by the manipulandum. The torque was associated with the moment of inertia of the object and the force was associated with the circular motion of the mass (τ and **F**, respectively in Fig 1B). Critically, the force caused the handle to displace unless subjects produced a compensatory force in the opposite direction (for full details about the object dynamics see [3]). Briefly, the force generated by rotating the object is dominated by the tangential acceleration of the mass and is approximately perpendicular to the rod. The direction of the force depends on the orientation of the object whereas its magnitude depends on the mass and the length of the rod. We have previously shown that subjects produce compensatory forces in the direction which is appropriate for the visual orientation of the object from the very first trial, even before they have experienced the dynamics in the task [24]. This is evidence that subjects have pre-existing knowledge (an internal model) of the dynamics of such objects. Moreover, subjects rapidly adapt the magnitude of these forces in order to minimise displacement of the handle [3].

A trial began with the handle stationary within the home region and the object aligned with the start target for that trial. The movement was cued by a tone and the appearance of the end target (40° from the start target). A successful trial ended when the subject had rotated the object to reach the end target. The end target then became the start target for the next trial (inset of Fig 1B). Subjects were required to make the movement within 400 ms, were warned if they took longer and had to repeat the trial if the movement exceeded 500 ms.

### Trial types

Subjects experienced the torque associated with rotating the object on all trials. However, the forces generated by the manipulandum could vary according to three different trial types. On exposure trials, subjects experienced the forces associated with rotating the object (that is, they experienced the full dynamics of the object). On error-clamp trials, the manipulandum simulated a stiff two-dimensional spring centred on the handle position at the start of the trial (the spring constant was 40 N/cm). Error-clamp trials effectively eliminate kinematic errors [51] and prevent error-driven adaptation. Error-clamp trials also allow the compensatory forces produced by subjects to be measured. Finally, on zero-force trials, the manipulandum did not produce any forces and the handle was free to move. Importantly, any forces produced by subjects on zero-force trials will cause the handle to displace.

### Experiments

Five experiments were performed. A partial analysis of experiments 1, 2 and 3 has been previously published and we include brief methods here (full details in [3,4,24]). All experiments began with a familiarization phase which consisted of repeated blocks of 12 zero-force trials. The blocks were presented at all orientations which subjects would experience during the particular experiment. Zero-force familiarization blocks for each orientation were repeated twice and presented in a pseudorandom order. This ensured that the force output produced by subjects prior to the experiment was low. Rest breaks (45 s) were given every 3–5 minutes in all experiments.

In Experiment 1, subjects (n = 12) first performed a block of 46 exposure trials at a single exposure orientation (E180°; Fig 3A and 3B). They were then presented with repeating probe blocks consisting of 20 error-clamp trials at one of five possible probe orientations (Pθ° relative to the exposure orientation, Pθ = {0°, 22.5°, 45°, 90°, 180°}). Each probe block was followed by a re-exposure block consisting 18 exposure trials with the object at the original exposure orientation (E180°). Each of the 5 probe block orientations was repeated 3 times in a pseudorandom order (total of 15 probe blocks). A second group (n = 12) performed the identical experiment except that they were exposed at 0° (E0°).

Experiment 2 was identical to Experiment 1 except that in this case the probe blocks consisted of 8 zero-force (de-adaptation) trials (Fig 3A). Again, separate groups of subjects (n = 12 in each group) were exposed to the object at 180° (E180°; Fig 3A and 3C) and 0° (E0°).

In Experiment 3, subjects (n = 12) performed 20 alternating blocks of 24 trials at 180° and 0° (10 blocks of each). The first 18 blocks were exposure trials (E180° and E0° in Fig 4A) and the last two block were zero-force trials (Z180° and Z0° in Fig 4A).

In Experiment 4, subjects (n = 12) performed four conditions in a randomized order (Fig 5A). In all conditions, the orientation of the object alternated across successive blocks (as in Experiment 3). All odd-numbered blocks were at 180° and all even-numbered blocks were either 0° (opposing dynamics condition; as in Experiment 3) or 270° (orthogonal dynamics condition as shown in Fig 5A). The first five blocks of the four conditions were always exposure blocks and the last two blocks were always zero-force blocks (Fig 5A). In the E6 condition, an additional exposure block at either 0° or 270° was performed before the final zero-force blocks. The four conditions thus comprised a combination of two factors: opposing versus orthogonal dynamics and exposure (E6) versus zero-force (Z6) trials on the 6th block.

In Experiment 5, subjects (n = 12) performed a block of 46 exposure trials with dynamics corresponding to an object at 180°. However, the rod and mass of the object were not displayed making the orientation visually ambiguous. To guide the appropriate rotations of the object, the object was displayed as a disc with small bars at 0° and 180° (see bottom inset of Fig 1B and exposure object in Fig 6A). As in the other experiments, these bars had to be aligned with the angular targets presented during the task. Importantly, the visual display of the ambiguous object still tracked the rotation and translation of the WristBOT handle. After the initial exposure block, subjects performed probe blocks of 20 error-clamp trials which alternating with re-exposure blocks of 18 exposure trials. Probe blocks were presented at one of two probe orientations (P0° and P180°; Fig 6A) and importantly, the normal visual object was displayed during the probes. During the re-exposure blocks, the visually ambiguous object was re-displayed. Each probe orientation was repeated 3 times in a pseudorandom order. A second group of subjects (n = 12) performed the identical experiment except that they were exposed to the visually ambiguous object with the dynamics at 0° (E0°; Fig 6C).

### Analysis

On zero-force and exposure trials, the peak displacement (PD) of the handle was measured, relative to its position at the start of the trial. Peak displacement is a measure of error, because the task required subjects to keep the handle as still as possible as they rotate the object. A PD of zero would thus indicate perfect performance. On error-clamp trials, the peak force produced by subjects was measured as the restoring force of the 2D simulated spring. Peak force is a measure of the subject’s adaptation to the object dynamics. Adaptation was calculated as the ratio of the peak force divided by the peak force that would fully compensate for the dynamics of the object on that trial. For example, at 0.5 adaptation the peak force produced by a subject during the rotation would be half the force generated by the object.

For experiments which included repeated blocks of probes trials (experiments 1, 2 and 5), we first averaged trial data for each subject across the repeats for each probe orientation and then averaged across subjects. We then created a composite trial-series consisting of the initial exposure trials (averaged across subjects), followed by the average probe blocks in order of increasing relative probe orientation (Δθ). These composite series were used for fitting the models and for presenting the data. Note that as the adaptive state is very similar at the start of each probe block, the order of the probe blocks in the composite trial series has minimal effect on the model parameters.

### The Error Tuned Model (ETM)

The Error Tuned Model (ETM) is a state-space model which implements a mixture-of-primitives modular architecture. Each module (or primitive) can produce force in only a single direction termed the module’s preferred direction (specified by *θ*_*i*_ for the *i*^*th*^ module). Therefore, each module can produce positive force in the direction of a 2-dimensional unit vector:

$$u_{i}=\left[\begin{array}{c} \cos\theta_{i}\\ \sin\theta_{i} \end{array}\right]$$ (4)

The *m* modules cover the range of possible force directions uniformly across 360° (in the two-dimensional task space). Each module is associated with a positive adaptive state ($x_{i}^{\left(n\right)}$ on the *n*^th^ trial) which can change over trials. The adaptive state represents the activity of that module. We represent the ensemble of preferred module directions as Θ_**x**_ = [*θ*_1_,…,*θ*_*m*_]^*T*^ and their adaptive states on trial *n* as $x^{\left(n\right)}={\left[x_{1}^{\left(n\right)},\ldots ,x_{m}^{\left(n\right)}\right]}^{T}$ (Fig 2A, left panel). On a given trial, the visual orientation of the object can be informative as to the direction of force required to compensate for the dynamics. We define $\theta_{v}^{\left(n\right)}$ as this force direction based on the visual orientation of the object and define the vector **Δ**^(*n*)^ as the difference between this angle and the preferred force directions of all the modules: $\Delta^{\left(n\right)}=\Theta_{x}-\theta_{v}^{\left(n\right)}$ (with the *i*^*th*^ element denoted by $\Delta_{i}^{\left(n\right)}$).

The net motor output (***z***^(*n*)^; Fig 2A, right panel) is a vector sum of force vectors weighted by both the adaptive states across modules and a contextual tuning function:

$$z^{\left(n\right)}=\sum_{i=1}^{m}C(\Delta_{i}^{\left(n\right)})\cdot x_{i}^{\left(n\right)}\cdot u_{i}$$ (5)

The contextual tuning function, $C(\Delta_{i}^{\left(n\right)})$, gives greater weights to modules whose preferred force direction is close to the direction that can compensate for the dynamics of the object, and progressively less weight as the preferred direction deviates (Fig 2A, middle panel). The contextual weight is represented as a Gaussian function of $\Delta_{i}^{\left(n\right)}$, with standard deviation *σ*_*c*_, scaled to have a maximum of 1 (at $\Delta_{i}^{\left(n\right)}=0$) and a minimum of *c*_180_ (at $\Delta_{i}^{\left(n\right)}=\pm 180$). Therefore, the net output is affected by both the adaptive states across modules as well as contextual information provided by vision of the object.

The adaptive states of the modules change across trials based on both error-independent decay and error-dependent adaptation:

$$x^{\left(n+1\right)}=\alpha (\Delta^{\left(n\right)})⊙x^{\left(n\right)}+\beta (\Delta^{\left(n\right)})⊙\cos(\Theta_{x}-\theta_{e}^{\left(n\right)})\cdot e^{\left(n\right)}$$ (6)

where ⊙ denotes element-wise vector multiplication. The first term specifies the error-independent decay (Fig 2B, top row) and is mediated by a context-dependent retention function ***α***(**Δ**^(*n*)^), whose elements determine the extent to which the adaptive state of each module is retained on the next trial (Fig 2B, top row, middle panel). This decay function is represented as a Gaussian function of **Δ**^(*n*)^ with standard deviation *σ*_*α*_, scaled to have values between *α*_0_ and *α*_180_, at $\Delta_{i}^{\left(n\right)}=0{}^{\circ}$ and $\Delta_{i}^{\left(n\right)}=\pm 180{}^{\circ}$, respectively.

The second term specifies the error-dependent adaptation (Fig 2B, bottom row) which updates the states based on the magnitude of the error (*e*^(*n*)^). The state update for each module in response to this error is dependent on two factors. First, the error is weighted by a visual context-dependent learning rate ***β***(**Δ**^(*n*)^) which is a Gaussian function of $\Delta_{i}^{\left(n\right)}$ with standard deviation *σ*_*β*_, scaled to have a value of *β*_0_ and *β*_180_, at $\Delta_{i}^{\left(n\right)}=0{}^{\circ}$ and $\Delta_{i}^{\left(n\right)}=\pm 180{}^{\circ}$, respectively (Fig 2B, bottom row, middle panel). Therefore, in general, the module which should be able to contribute most to reduce error (based on the visual orientation of the object) is updated the most. The second factor modulating adaptation depends on the direction of the kinematic error. In the model, each module can only reduce errors along its preferred force direction by increasing or decreasing its adaptive state. Therefore, the adaptive state of each module is changed in proportion to the component of error projected onto its preferred force direction. This projection is calculated by multiplying the magnitude of the error (*e*^(*n*)^) by the cosine of the angular difference between the error direction ($\theta_{e}^{\left(n\right)}$) and the preferred force direction for each module (Fig 2B, bottom row, left panel).

Without loss of generality, we define the force generated by the object on exposure trials to have unit magnitude. The kinematic error is determined by the discrepancy between the force output of the model (which represents the force produced by the subject) and the force produced by the object. The error magnitude is also modulated by a compliance-dependent function. The error also depends on whether the trial is an exposure trial (*p* = 1) or a zero-force trial (*p* = 0):

$$e^{\left(n\right)}=f_{\theta_{v}^{\left(n\right)},p^{\left(n\right)}}\cdot (p^{\left(n\right)}\left[\begin{array}{c} \cos\theta_{v}^{\left(n\right)}\\ \sin\theta_{v}^{\left(n\right)} \end{array}\right]-z^{\left(n\right)})$$ (7)

Here, $f_{\theta_{v}^{\left(n\right)},p^{\left(n\right)}}$ linearly maps state errors in the model to the magnitude of kinematic errors and is specified separately for each object orientation and trial type (zero-force and exposure). The error vector magnitude (*e*^(*n*)^) and angle ($\theta_{e}^{\left(n\right)}$) are then used to update the adaptive states across modules (Eq 6). On error-clamp trials *e*^(*n*)^ = **0**.

### Model fitting

The free parameters of the ETM model include: *c*_180_, *σ*_*c*_, *β*_0_, *β*_180_, *σ*_*β*_, *α*_0_, *α*_180_, and *σ*_*α*_. In the fits and simulations, we used *m* = 16 modules. We set the standard deviations for the Gaussian functions tuning functions $C(\Delta_{i}^{\left(n\right)})$ and ***β***(**Δ**^(*n*)^) to be equal (*σ*_*c*_ = *σ*_*β*_ = *σ*), both to reduce the degrees of freedom in the model and because the experimental paradigms were not designed to constrain each of these parameters separately. The number of free parameters in the ETM was thus 7. The compliance-dependent error function ($f_{\theta_{v}^{\left(n\right)},p^{\left(n\right)}}$) has been previously estimated for this task and we used these values [3].

There are two measures from the experimental trial-series. On zero-force and exposure trials we measure peak displacement whereas on error-clamp trials we measure adaptation. To use all the trials to fit the model we need to represent them in the same units. Therefore, for error-clamp trials we converted both the adaption measured on that trial, as well as the motor output predicted by the model, to the equivalent kinematic error using Eq 7. For each experiment we averaged the trial data across subjects and then fit by minimizing the mean squared error between the empirical and predicted kinematic errors.

The 7 parameter ETM was fit simultaneously to the trial-series from experiments 1, 2 and 3. The trial-series were obtained from 5 different groups of subjects (2 groups for the 2 conditions in Experiment 1, 2 groups for the 2 conditions in Experiment 2, and 1 group for Experiment 3). Experiments 4 and 5 were also fit simultaneously. Because experiments 4 and 5 only use a limited number of object orientations (no more than three) they were not able to constrain the *σ* and *σ*_*c*_ parameters of the Gaussian tuning functions. We therefore fixed these parameters to the values obtained from fitting the first three experiments. In Experiment 5, we used a visually ambiguous object. On the trials where the visually ambiguous object was presented, the context-dependent tuning functions $C(\Delta_{i}^{\left(n\right)})$, ***α***(**Δ**^(*n*)^) and ***β***(**Δ**^(*n*)^), were set to be constant across all orientations. This required three additional parameters: *c*_*a*_, *α*_*a*_ and *β*_*a*_.

To account for differences in the stiffness and other biomechanical properties across the groups of subjects, we also included parameters that allowed the peak displacement and adaptation measures to be related linearly to the model predictions for each subject group. Importantly, because these additional parameters can only scale model output uniformly within each group of subjects, they do not qualitatively influence the predictions of the model (which are always tested within each group).

### Modelling object lifting

We also fit the ETM and CDM to results from a previous study [23] in which subjects lifted a physical object using handles (grasp points) either on the left or right side of the object (Fig 7A). Subjects were asked to lift the object while minimizing tilt. This required them to generate compensatory torques which depended on the location of the centre of mass relative to the grasp point.

In the case of object lifting, each module in the ETM can generate a torque around a single fixed preferred oriented axis (with torque direction specified by the right-hand rule). The context is the orientation of the centre of mass relative to the grasp point. In general, the preferred axes across modules would uniformly cover all orientations in the horizontal plane (corresponding to *θ*_*i*_ in the ETM). However, as this previous study used only two orientations (two grasp points), we fit a simplified version of the ETM which has two modules, *θ*_*i*_ ∈ {0°, 180°}. These modules correspond to producing CCW and CW torques for the left and right grasp points. In this case, the ETM generates torques aimed at minimising object tilt (instead of forces aimed at minimising object displacement).

The ETM with only two modules reduces the degrees of freedom of the model from 7 to 5 (because with only two orientations in the experiment, the *σ*_*α*_ and *σ* parameters for the contextual tuning functions are not fit). For simplicity, we also set the retention factor for the non-preferred module (*α*_180_) to 1. Because subjects have an initial estimate of the torque required to lift objects in this task (for example, based on visual cues), we assumed that the adaptive state of both modules (*x*°) was initially non-zero and we also fit this parameter. We fit absolute error from the model (*e*^(*n*)^) to the absolute peak object tilt from the experiment. We included an additional fit parameter (*k*) which scaled the motor output to units of peak roll. After fitting the model, we performed a linear regression to map the output of the model (*z*) to the experimental torque data (see Fig 7B). The same process was repeated for fitting the CDM.

### The Context-Dependent Decay Model (CDM)

For comparison with the ETM, we also include fits of our previous context-dependent decay model (CDM; [4]). The CDM differs from the ETM in two critical aspects. First, the CDM describes the motor output and errors as signed scalar values rather and 2D vectors. The scalar value for motor output in the CDM represents only the magnitude of the force required to compensate for the object dynamics and in this case is simply the adaptive state of the currently selected module. The sign of the scalar error indicates whether the motor output on a given trial is too small (positive errors) or too large (negative errors) to compensate for the object dynamics on that trial. Second, because errors are signed scalars in the CDM, there is no tuning for the direction of error. In contrast, in the ETM, tuning for the direction of error is critical to the model and is implemented by the cosine term in Eq 6. Despite the differences between the current ETM and previous CDM, the equations for the CDM can be expressed using similar notation. The scalar motor output in the CDM is:

$$z^{\left(n\right)}=\sum_{i=1}^{m}C(\Delta_{i}^{\left(n\right)})\cdot x_{i}^{\left(n\right)}$$ (8)

where, $C(\Delta_{i}^{\left(n\right)})$ is now a binary selection function that is 1 for module *i* when $\Delta_{i}^{\left(n\right)}=0$, and is 0 otherwise. That is, only the module whose preferred direction matches the orientation of the object contributes to the motor output. The state-update equation for the CDM is:

$$x^{\left(n+1\right)}=\alpha (\Delta^{\left(n\right)})⊙x^{\left(n\right)}+\beta (\Delta^{\left(n\right)})\cdot e^{\left(n\right)}$$ (9)

Where, the functions ***α***(**Δ**^(*n*)^) and ***β***(**Δ**^(*n*)^) are the retention and learning-rate functions, respectively (as defined in the ETM). Finally, the error in the CDM is a signed scalar:

$$e^{\left(n\right)}=f_{\theta_{v}^{\left(n\right)},p^{\left(n\right)}}\cdot (p^{\left(n\right)}-z^{\left(n\right)})$$ (10)

Where, $f_{\theta_{v}^{\left(n\right)},p^{\left(n\right)}}$ and *p*^(*n*)^ is the compliance-dependent error function and the trial-type indicator, respectively (as in Eq 7 for the ETM). The CDM therefore has 6 parameters (the same parameters as the ETM, excluding *c*_180_).

### Model comparison

Model selection between the ETM and CDM was performed using the Bayesian Information Criterion (BIC). The BIC for a particular model combines a ‘‘reward” for the goodness of fit with a ‘‘penalty” for the number of free parameters:

$$BIC=N\cdot \ln(\sigma_{e}^{2})+k\cdot \ln(N)$$ (11)

where $\sigma_{e}^{2}$ is the variance in the residual errors of the fit, *k* is the number of free parameters and *N* is the number of data points (the number of trials). Taking the difference in BIC values for two competing models approximates half the log of the Bayes factor [52]. A BIC difference of greater than 4.6 (a Bayes factor of greater than 10) is considered to provide strong evidence in favour of the model with the lower BIC value [53].

### Bootstrap analysis

Confidence limits for the model fits, model parameters and R^2^ values were calculated using a bootstrap analysis [3,26]. Specifically, 10,000 unique samples of 12 subjects were drawn with replacement from the subject pool for each experiment (n = 12 subjects in all experiments). The models were fit separately to the mean of each bootstrap sample, as described above. The 95% confidence limits were calculated as the 2.5 and 97.5 percentile values from the distributions for the model fits, model parameters and R^2^ values across the 10,000 samples. P-values for the BIC model selection were calculated as the proportion of samples with BIC values in favor of the selected model.

The 95% confidence limits for the model fits across the 5 experiments are shown as shaded error-bars in the Supporting S2 and S3 Figs. The 95% confidence limits for model parameters and R^2^ values, and the p-values for BIC model selection are presented in Supporting S1 Table.

### Optimality of cosine error tuning

In addition to model fitting and simulations, we also show that cosine tuning for errors in the state-update function (adaptation) is optimal in reducing the error with the minimal change in the adaptive states of the modules (akin to the minimum intervention principle). For simplicity, we consider the update rule in the absence of both contextual information and error-independent decay.

Consider *m* modules with adaptive states **x** = [*x*_1_,…,*x*_*m*_]^*T*^. The preferred direction of each module is given by *θ*_*i*_ (for the *i*^*th*^ module) and each module can produce positive forces in the direction of a 2-dimensional force vector ***u***_*i*_ = [cos*θ*_*i*_, sin*θ*_*i*_]^*T*^. The net motor output is the sum of the force vectors weighted by the adaptive states:

$$z=\sum_{i=1}^{m}x_{i}\cdot u_{i}=\mathrm{Ux}$$ (12)

where, **U** is the ensemble of force vectors over modules:

$$U={\left[\begin{array}{ccc} \cos\theta_{1} & \cdots & \cos\theta_{m}\\ \sin\theta_{1} & \cdots & \sin\theta_{m} \end{array}\right]}_{2\times m}$$ (13)

We consider a trial-series in which a constant perturbation (***f***) is applied and consider the change in the adaptive state of the modules between two consecutive trials (from **x**^(*n*)^ to **x**^(*n*+1)^). The perturbation on trial *n* gives rise to an error vector ***e***^(*n*)^ = ***f***−***z***^(*n*)^, which we express in terms of a magnitude (*e*^(*n*)^) and direction (*θ*_*e*_):

$$e^{\left(n\right)}=e^{\left(n\right)}\left[\begin{array}{c} \cos\theta_{e}\\ \sin\theta_{e} \end{array}\right]$$ (14)

We assume an adaptation mechanism that updates the adaptive states to correct for the error:

$$x^{\left(n+1\right)}=x^{\left(n\right)}+\beta \cdot e^{\left(n\right)}\cdot g$$ (15)

where *β* is the learning rate, and g is a tuning vector whose elements determine how each module updates in response to the error. After the state update, the error on the next trial is given by:

$$e^{\left(n+1\right)}=f-z^{\left(n+1\right)}=f-Ux^{\left(n+1\right)}=f-U(x^{\left(n\right)}+\beta \cdot e^{\left(n\right)}\cdot g)$$ (16)

Applying ***z***^(*n*)^ = **Ux**^(*n*)^ and ***e***^(*n*)^ = ***f***−***z***^(*n*)^ in the above expression gives:

$$e^{\left(n+1\right)}=e^{\left(n\right)}-\beta \cdot e^{\left(n\right)}\cdot \mathrm{Ug}$$ (17)

The goal is to find the vector g such that **Ug** acts to directly reduce the error. Specifically, **Ug** is a vector in the opposite direction to ***e***^(*n*)^ which is therefore capable of reducing the error to zero over multiple trials. To find g we solve for the update that would reduce the error to zero (***e***^(*n*+1)^ = **0**) in one trial (setting *β* = 1). Note that in practice, however, *β* is less than 1, and modulates the proportion of the error that is actually corrected on a single trial. To find g with *β* = 1 from Eq 17, we have:

$$e^{\left(n\right)}-e^{\left(n\right)}\cdot \mathrm{Ug}=0$$

$$\Rightarrow \mathrm{Ug}=\frac{e^{\left(n\right)}}{e^{\left(n\right)}}\Rightarrow \mathrm{Ug}=\left[\begin{array}{c} \cos\theta_{e}\\ \sin\theta_{e} \end{array}\right]$$ (18)

Defining *B* ≔ [cos*θ*_*e*_, sin*θ*_*e*_]^*T*^, the above equation has a simple form:

Ug=B (19)

However, it is under-constrained as it involves two equations with *m* unknowns (*m* elements of g). Here we show that cosine tuning is the optimal solution as it allows minimum intervention across the activity of modules. Specifically, cosine tuning minimizes the L-2 norm of vector g (‖g‖). Therefore, the optimization problem is to find g such that:

g=argmin(‖g‖)

givenUg=B (20)

The above problem is a standard least-norm optimization problem that has the following analytical solution [54]:

$$g=U^{T}{\left(UU^{T}\right)}^{-1}B$$ (21)

The term **UU**^*T*^ is given by:

$$UU^{T}=\left[\begin{array}{cc} \sum_{i=1}^{m}\cos^{2}\theta_{i} & \frac{1}{2}\sum_{i=1}^{m}\sin2\theta_{i}\\ \frac{1}{2}\sum_{i=1}^{m}\sin2\theta_{i} & \sum_{i=1}^{m}\sin^{2}\theta_{i} \end{array}\right]$$ (22)

Because the preferred module angles (*θ*_*i*_) are uniformly distributed across the unit circle (*θ*_*i*_ = 2π(*i*/*m*), *i* = 1,…,*m*), it is simple to show that:

$$\sum_{i=1}^{m}\sin k\theta_{i}=0,\;\sum_{i=1}^{m}\cos k\theta_{i}=0$$ (23)

for *k* = 1,2,… (and any integer that is not divisible by *m*). Using this property, the off-diagonal elements of the matrix **UU**^*T*^ are equal to 0 (here, *k* = 2). Also, using trigonometric transformations, the diagonal elements are obtained as follows:

$$\sum_{i=1}^{m}\cos^{2}\theta_{i}=\frac{1}{2}\sum_{i=1}^{m}\left(1-\cos2\theta_{i}\right)=\frac{m}{2}+\frac{1}{2}\sum_{i=1}^{m}\cos2\theta_{i}=\frac{m}{2}$$

$$\sum_{i=1}^{m}\sin^{2}\theta_{i}=\frac{1}{2}\sum_{i=1}^{m}\left(1-\cos2\theta_{i}\right)=\frac{m}{2}-\frac{1}{2}\sum_{i=1}^{m}\cos2\theta_{i}=\frac{m}{2}$$ (24)

The above leads to:

$$UU^{T}=\frac{m}{2}\left[\begin{array}{cc} 1 & 0\\ 0 & 1 \end{array}\right],{\left(UU^{T}\right)}^{-1}=\frac{2}{m}\left[\begin{array}{cc} 1 & 0\\ 0 & 1 \end{array}\right]$$ (25)

Therefore, Eq 21 can now be simplified as follows:

$$g=U^{T}{\left(UU^{T}\right)}^{-1}B=(2/m)U^{T}B$$ (26)

By performing the above multiplication, given the matrices **U** and *B* defined earlier, we obtain:

$$g=\frac{2}{m}\left[\begin{array}{c} \cos\theta_{1}\cos\theta_{e}+\sin\theta_{1}\sin\theta_{e}\\ \vdots \\ \cos\theta_{m}\cos\theta_{e}+\sin\theta_{m}\sin\theta_{e} \end{array}\right]=\frac{2}{m}\left[\begin{array}{c} \cos(\theta_{1}-\theta_{e})\\ \vdots \\ \cos(\theta_{m}-\theta_{e}) \end{array}\right]$$ (27)

Finally, by defining *w* = 2/*m*, and introducing the vector of preferred orientations across modules as Θ = [*θ*_1_,…,*θ*_*m*_]^*T*^, we can rewrite the solution in Eq 27 as follows:

$$g=w\cdot \cos(\Theta -\theta_{e})$$ (28)

As shown, the optimal solution provided in Eq 21 for minimizing the intervention across modules is a cosine tuning function that projects the error direction (*θ*_*e*_) onto the preferred module directions (Θ). The cosine tuning for error direction in the ETM is therefore optimal under these conditions.

## Supporting information

S1 Table Model parameters and 95% confidence limits. Parameters for the CDM and ETM when fit to datasets obtained from the different experiments. In the first dataset (top 2 main rows for CDM and ETM), experiments 1, 2 and 3 were concurrently fit with all free model parameters. In the second dataset (bottom 2 main rows for CDM and ETM), experiments 4 and 5 were concurrently fit with the Gaussian tuning function widths fixed to values obtained from fitting the first dataset (grey backgrounds indicate the fixed tuning-width values). BIC values are relative to the best model (the ETM in both cases) within the fits for each dataset. The 95% confidence limits (CL) on parameters and R^2^ values were calculated from a bootstrap analysis (see Methods of the main text). P-values for model selection were calculated as the proportion of bootstrap samples in which the BIC selected the ETM (see Methods and Table 1 in the main text for more details).
(PDF)

S1 Fig Experiments 1 and 2 (exposure at 0°). **A.** The paradigm for experiments 1 and 2 (E0° condition). After an initial exposure block at 0° (yellow background), subjects performed alternating probe blocks presented at one of five orientations between 0° and 180° (green background) followed by re-exposure blocks at 0° (blue background). **B.** Experiment 1 in which probe blocks consisted of 20 error-clamp trials. The left plot shows the composite trial series for PD (all trials) and Adaptation (error-clamp probe blocks only). Grey shading shows ±SE across subjects. The right plots show the corresponding measures averaged over the different probe blocks and over subjects (error-bars show ±SE across subjects). See Fig 3B in the main text for more details. **C.** Experiment 2, plotted as in panel B. In this case, probe blocks consisted of 8 zero-force trials. See Fig 3C in the main text for more details.
(PDF)

S2 Fig Experiments 1, 2 and 3 (95% confidence limits on model fits). Trial-series plots show experimental data (black) and fits for the error-tuned model (ETM) with 95% confidence limits (red line with pink shading) obtained from a bootstrap analysis (see main text for details). **A, B.** Experiment 1 (E180° and E0° conditions; see Fig 3B in the main text for details). **C, D.** Experiment 2 (E180° and E0° conditions; see Fig 3C in the main text for details). **E.** Experiment 3 (see Fig 4B in the main text for details).
(PDF)

S3 Fig Experiments 4 and 5 (95% confidence limits on model fits). Trial-series plots show experimental data (black) and fits for the error-tuned model (ETM) with 95% confidence limits (red line with pink shading) obtained from a bootstrap analysis (see main text for details). **A.** Experiment 4 (see Fig 5B in the main text for details). **B, C.** Experiment 5 (E180° and E0° conditions; see Fig 6B and 6C in the main text for details).
(PDF)

S1 Data Experimental data. Data for all experiments, conditions and subjects (Matlab MAT-file and m-file description) are available in the ZIP-file.
(ZIP)
