# Supplementary material for: An error-tuned model for sensorimotor learning
Source: PLoS Comput Biol. 2017 Dec 18;13(12):e1005883. doi: 10.1371/journal.pcbi.1005883 (PMC5749863; doi:10.1371/journal.pcbi.1005883)
Supplement: S1 Table — Parameters for the CDM and ETM when fit to datasets obtained from the different experiments. In the first dataset (top 2 main rows for CDM and ETM), experiments 1, 2 and 3 were concurrently fit with all free model parameters. In the second dataset (bottom 2 main rows for CDM and ETM), experiments 4 and 5 were concurrently fit with the Gaussian tuning function widths fixed to values obtained from fitting the first dataset (grey backgrounds indicate the fixed tuning-width values). BIC values are relative to the best model (the ETM in both cases) within the fits for each dataset. The 95% confidence limits (CL) on parameters and R2 values were calculated from a bootstrap analysis (see Methods of the main text). P-values for model selection were calculated as the proportion of bootstrap samples in which the BIC selected the ETM (see Methods and Table 1 in the main text for more details). (PDF) [file pcbi.1005883.s001.pdf]

Supporting Table S1 – Model Parameters and 95% Confidence Limits

Parameters for the CDM and ETM when fit to datasets obtained from the different experiments. In the first dataset (top 2 main rows for CDM and ETM), experiments 1, 2 and 3 were concurrently fit with all free model parameters. In the second dataset (bottom 2 main rows for CDM and ETM), experiments 4 and 5 were concurrently fit with the Gaussian tuning function widths fixed to values obtained from fitting the first dataset (grey backgrounds indicate the fixed tuning-width values). BIC values are relative to the best model (the ETM in both cases) within the fits for each dataset. The 95% confidence limits (CL) on parameters and R^2 values were calculated from a bootstrap analysis (see Methods of the main text). P-values for model selection were calculated as the proportion of bootstrap samples in which the BIC selected the ETM (see Methods and Table 1 of the main text for more details).

Model	Exp's	DoF	α_0	α_{180}	β_0	β_{180}	σ_α	σ	C_{180}	α_a	β_a	C_a	R^2	ΔBIC
CDM	1,2,3	6	0.9857	0.9981	0.1429	0.0001	50.8	31.1	-	-	-	-	0.87	122.0
95% CL			0.9810	0.9947	0.1031	0.0001	33.3	25.4	-	-	-	-	0.75	-
			0.9899	0.9999	0.1817	0.0225	71.5	49.3					0.86	
ETM	1,2,3	7	0.9783	0.9960	0.1095	0.0225	38.9	14.5	0.0323	-	-	-	0.88	0.0
95% CL			0.9512	0.9929	0.0622	0.0001	8.1	11.7	0.0000	-	-	-	0.77	p=0.0150
			0.9815	0.9983	0.1563	0.0593	58.5	39.9	0.2449				0.87	
CDM	4,5	7	0.9731	0.9888	0.1826	0.0001	50.8	31.1	-	0.9895	0.0130	0.3769	0.88	287.3
95% CL			0.9538	0.9851	0.0887	0.0001	-	-	-	0.9800	0.0109	0.3369	0.75	-
			0.9925	0.9918	0.2535	0.0005				0.9969	0.0240	0.3790	0.86	
ETM	4,5	8	0.9720	0.9957	0.1659	0.0222	38.9	14.5	0.1011	0.9895	0.0131	0.4285	0.90	0.0
95% CL			0.9517	0.9914	0.0990	0.0058	-	-	0.0410	0.9851	0.0100	0.3847	0.78	p<0.0001
			0.9881	0.9999	0.2103	0.0466			0.1739	0.9965	0.0860	0.4924	0.88	
