# Supplementary material for: An error-tuned model for sensorimotor learning
Source: PLoS Comput Biol. 2017 Dec 18;13(12):e1005883. doi: 10.1371/journal.pcbi.1005883 (PMC5749863; doi:10.1371/journal.pcbi.1005883)

Supporting Figure S1 – Experiments 1 and 2 (Exposure at 0°)

A. The paradigm for experiments 1 and 2 (E0° condition). After an initial exposure block at 0° (yellow background), subjects performed alternating probe blocks presented at one of five orientations between 0° and 180° (green background) followed by re-exposure blocks at 0° (blue background). **B.** Experiment 1 in which probe blocks consisted of 20 error-clamp trials. The left plot shows the composite trial series for PD (all trials) and Adaptation (error-clamp probe blocks only). Grey shading shows ±SE across subjects. The right plots show the corresponding measures averaged over the different probe blocks and over subjects (error-bars show ±SE across subjects). See Figure 3B in the main text for more details. **C.** Experiment 2, plotted as in panel B. In this case, probe blocks consisted of 8 zero-force trials. See Figure 3C in the main text for more details.

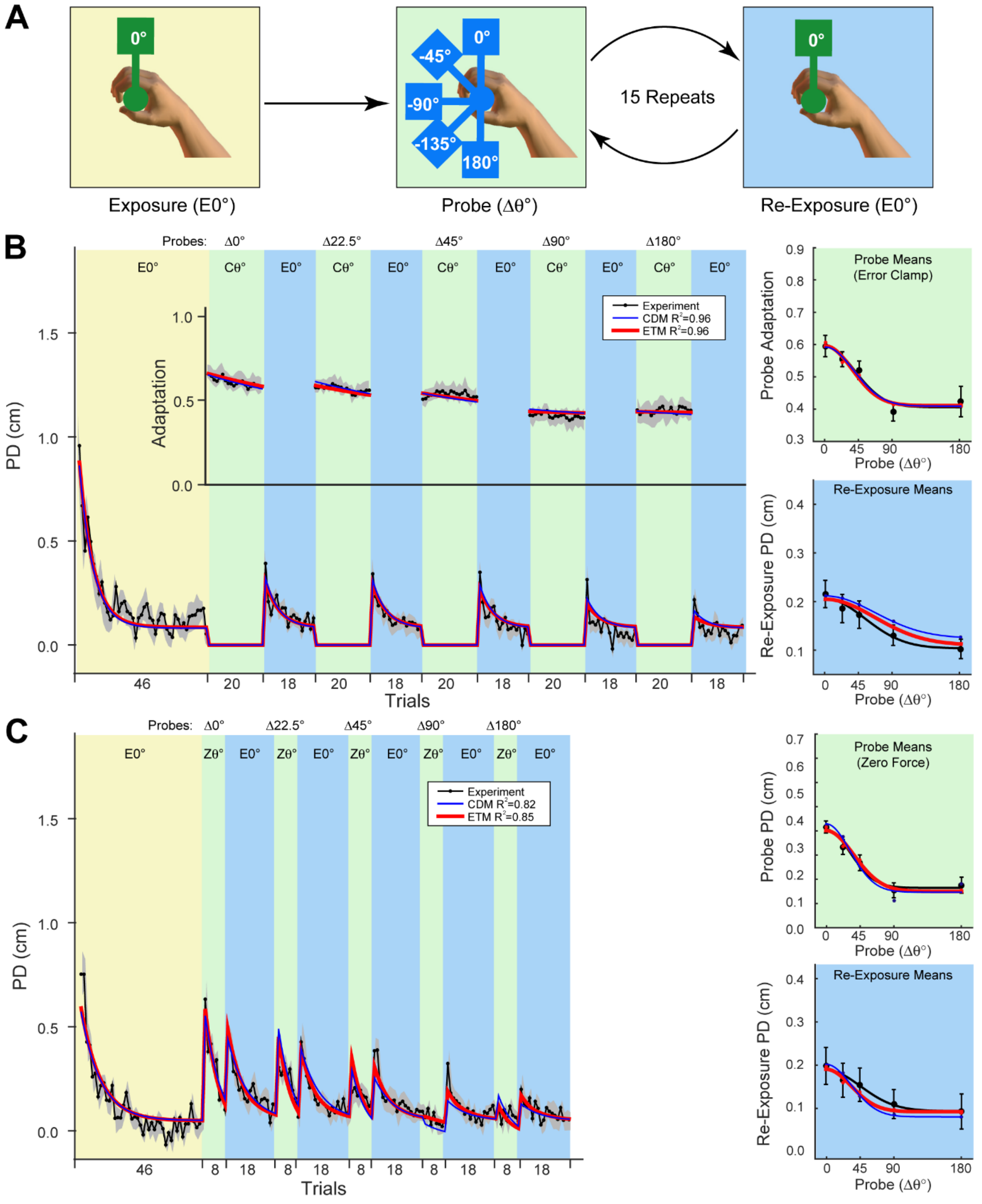

Supplement: S1 Fig — A. The paradigm for experiments 1 and 2 (E0° condition). After an initial exposure block at 0° (yellow background), subjects performed alternating probe blocks presented at one of five orientations between 0° and 180° (green background) followed by re-exposure blocks at 0° (blue background). B. Experiment 1 in which probe blocks consisted of 20 error-clamp trials. The left plot shows the composite trial series for PD (all trials) and Adaptation (error-clamp probe blocks only). Grey shading shows ±SE across subjects. The right plots show the corresponding measures averaged over the different probe blocks and over subjects (error-bars show ±SE across subjects). See Fig 3B in the main text for more details. C. Experiment 2, plotted as in panel B. In this case, probe blocks consisted of 8 zero-force trials. See Fig 3C in the main text for more details. (PDF) [file pcbi.1005883.s002.pdf]
