# Supplementary material for: An error-tuned model for sensorimotor learning
Source: PLoS Comput Biol. 2017 Dec 18;13(12):e1005883. doi: 10.1371/journal.pcbi.1005883 (PMC5749863; doi:10.1371/journal.pcbi.1005883)

**Supporting Figure S2 – Experiments 1, 2 and 3 (95% Confidence Limits on Model Fits)**

Trial-series plots show experimental data (black) and fits for the error-tuned model (ETM) with 95% confidence limits (red line with pink shading) obtained from a bootstrap analysis (see main text for details). **A, B.** Experiment 1 (E180° and E0° conditions; see Figure 3B in the main text for details). **C, D.** Experiment 2 (E180° and E0° conditions; see Figure 3C in the main text for details). **E.** Experiment 3 (see Figure 4B in the main text for details).

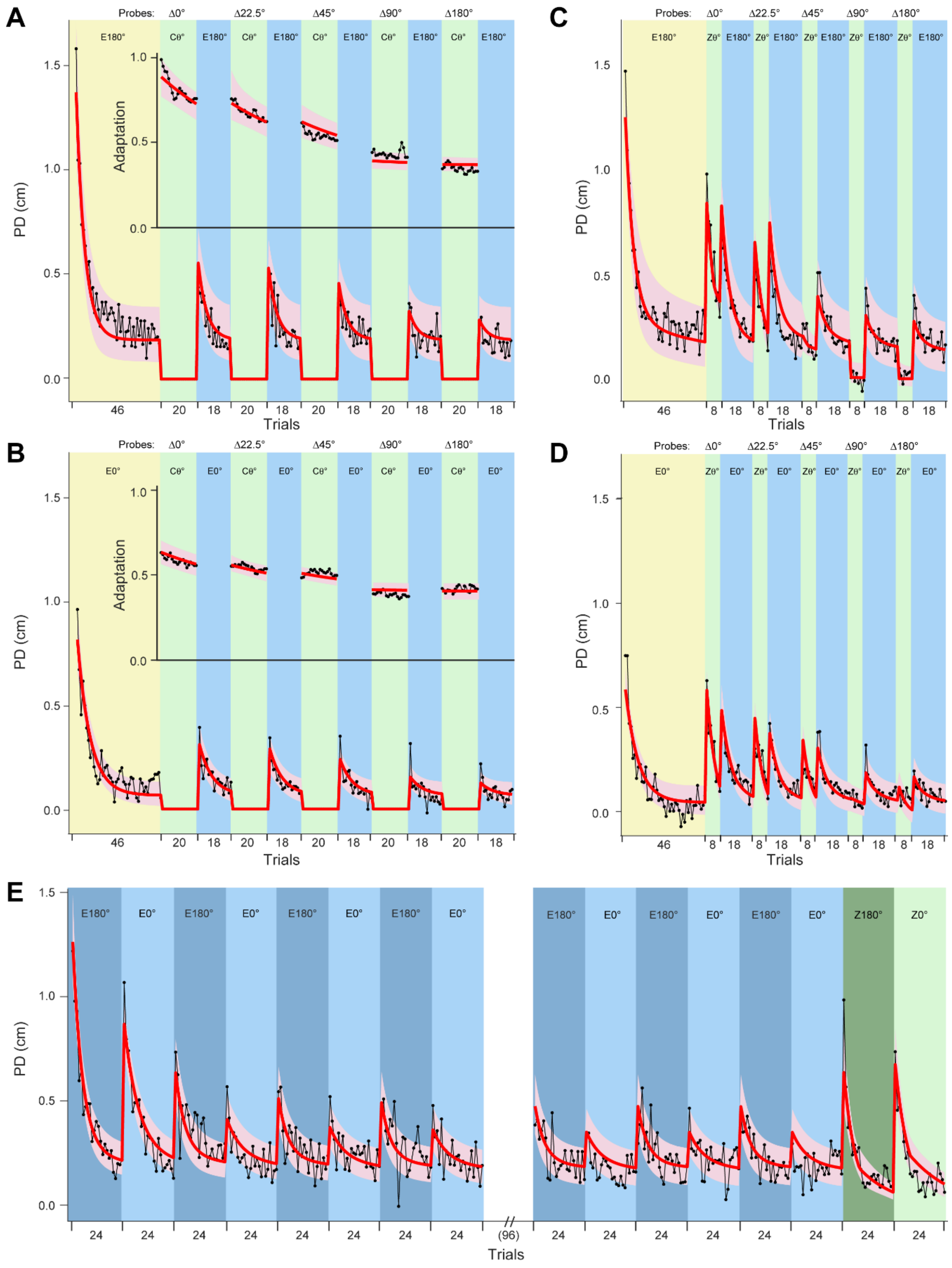

Supplement: S2 Fig — Trial-series plots show experimental data (black) and fits for the error-tuned model (ETM) with 95% confidence limits (red line with pink shading) obtained from a bootstrap analysis (see main text for details). A, B. Experiment 1 (E180° and E0° conditions; see Fig 3B in the main text for details). C, D. Experiment 2 (E180° and E0° conditions; see Fig 3C in the main text for details). E. Experiment 3 (see Fig 4B in the main text for details). (PDF) [file pcbi.1005883.s003.pdf]
