# Supplementary material for: An error-tuned model for sensorimotor learning
Source: PLoS Comput Biol. 2017 Dec 18;13(12):e1005883. doi: 10.1371/journal.pcbi.1005883 (PMC5749863; doi:10.1371/journal.pcbi.1005883)

Supporting Figure S3 – Experiments 4 and 5 (95% Confidence Limits on Model Fits)

Trial-series plots show experimental data (black) and fits for the error-tuned model (ETM) with 95% confidence limits (red line with pink shading) obtained from a bootstrap analysis (see main text for details). **A.** Experiment 4 (see Figure 5B in the main text for details). **B, C.** Experiment 5 (E180° and E0° conditions; see Figure 6B and 6C in the main text for details).

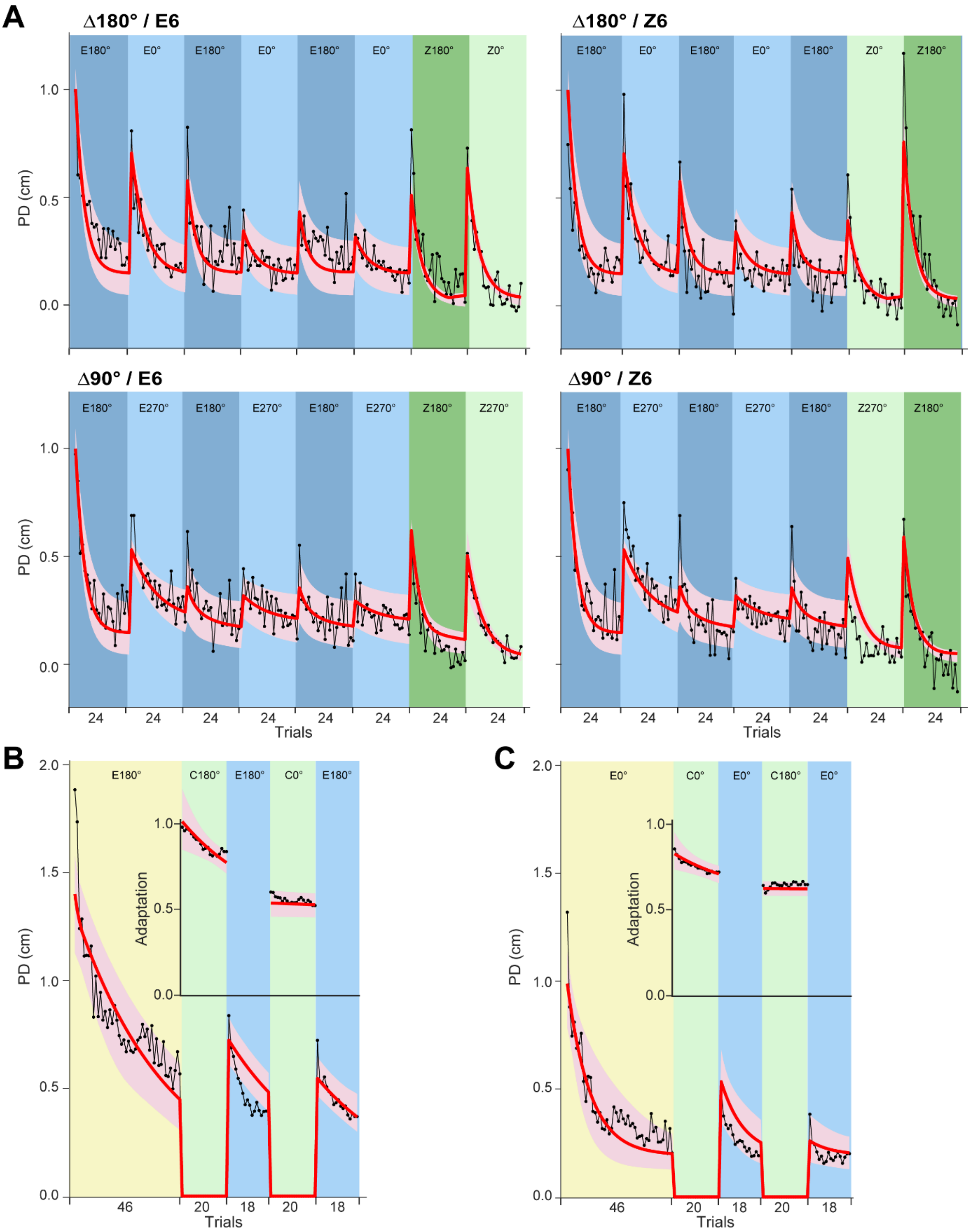

Supplement: S3 Fig — Trial-series plots show experimental data (black) and fits for the error-tuned model (ETM) with 95% confidence limits (red line with pink shading) obtained from a bootstrap analysis (see main text for details). A. Experiment 4 (see Fig 5B in the main text for details). B, C. Experiment 5 (E180° and E0° conditions; see Fig 6B and 6C in the main text for details). (PDF) [file pcbi.1005883.s004.pdf]
